# The Prevalence of Self-injurious Behaviour in Autism: A Meta-analytic Study

**DOI:** 10.1007/s10803-020-04443-1

**Published:** 2020-04-15

**Authors:** Catherine Steenfeldt-Kristensen, Chris A. Jones, Caroline Richards

**Affiliations:** 1 School of Psychology, University of Birmingham, Edgbaston, Birmingham, B15 2TT UK; 2 Children’s Neurodevelopmental Service, Coventry and Warwickshire Partnership Trust, City of Coventry Health Centre, Paybody Building, 2 Stoney Stanton Road, Coventry, CV1 4FS UK

**Keywords:** Autism, Self-injurious behaviour, Prevalence, Intellectual disability, Self-harm

## Abstract

Self-injurious behaviour is purportedly common in autism, but prevalence rates have not yet been synthesised meta-analytically. In the present study, data from 14,379 participants in thirty-seven papers were analysed to generate a pooled prevalence estimate of self-injury in autism of 42% (confidence intervals 0.38–0.47). Hand-hitting topography was the most common form of self-injury (23%), self-cutting topography the least common (3%). Sub-group analyses revealed no association between study quality, participant intellectual disability or age and overall prevalence rate of self-injury. However, females obtained higher prevalence rates than males (*p* = .013) and hair pulling and self-scratching were associated with intellectual disability (*p* = .008 and *p* = .002, respectively). The results confirm very high rates of self-injury in autism and highlight within group risk-markers.

**Electronic supplementary material:**

The online version of this article (10.1007/s10803-020-04443-1) contains supplementary material, which is available to authorized users.

## Introduction

The prevalence of autism within the general population varies, with recent population estimates ranging from 1 in 100 (Baird et al. 2006; Centre for Disease Control 2009) to 1 in 68 (Centre for Disease Control 2014) and higher rates of autism reported in males than in females (Baird et al. 2006; Fombonne 2009; Saracino et al. 2010). It is well established that the consequences and co-morbidities of autism pose a significant challenge for individuals, families and services (Altiere and von Kluge 2009; Hastings and Brown 2002; Hastings 2003; Knapp et al. 2009). For example, individuals with autism are at a greater risk of having co-morbid medical and psychiatric conditions such as sleep disorders (Mannion et al. 2013), mental health problems (Bradley et al. 2004; Brereton et al. 2006; Ghaziuddin et al. 1992), and intellectual disability (Matson and Shoemaker 2009). In particular, the high prevalence of co-morbid intellectual disability in those with autism (Matson and Shoemaker 2009) means that some individuals require specialist care and support throughout their lifetime. Given the substantial impact on quality of life for those with autism, it is important to further our understanding of the factors that contribute most significantly to the development and consequences of co-morbid disorders in autism, such as symptom severity, presence and/or severity of intellectual disability, age and gender.

In addition to co-morbid intellectual disability and psychiatric diagnoses, the presence of behaviours that challenge (i.e. aggression, destruction and self-injurious behaviour) in individuals with autism have a significant impact on quality of life (Baghdadli et al. 2003; Duerden et al. 2012; McTierman et al. 2011; Moyal et al. 2014; Murphy et al. 2009; Rattaz et al. 2015; Richards et al. 2012). In particular, research highlights that those with autism display more behaviours that challenge than their typically developing peers (McClintock et al. 2003), than those with a psychiatric diagnosis only (Matson et al. 2009) and than those with intellectual disability of heterogeneous aetiology (Davies and Oliver 2013; Holden and Gitlesen 2006; McClintock et al. 2003; Oliver, Murphy and Corbett 1987).

Notably, self-injurious behaviour is particularly common in individuals with autism (Matson et al. 2009). Self-injurious behaviour refers to a series of aggressive behaviours that an individual directs towards themselves that have the potential to result in physical injury, typically in the form of tissue damage (Matson and Turygin 2012). Common forms of self-injurious behaviour include head banging, self-biting, skin scratching, hair pulling and hitting oneself against hard objects (Cooper et al. 2009; Iwata et al. 1994; Minshawi et al. 2014; Weiss 2002). The presence of self-injurious behaviour has been associated with higher rates of psychiatric hospitalisation (Mandell 2008), more reactive physical interventions (Allen et al. 2009), lower quality of life (Beadle-Brown et al. 2009), exclusion from mainstream services (Knapp et al. 2005), and is the primary cause of emergency room visits among children with autism (Kalb et al. 2012). Furthermore, the detrimental impact of self-injurious behaviour extends beyond the individual with consequences for the whole family. For example, quality of life has been found to be lower in parents of children with autism due to heightened rates of behavioural problems, most notably, self-injurious behaviour (Baghdadli et al. 2014; Greenberg et al. 2006; LeCavalier et al. 2006). Finally, a recent study demonstrated that self-injurious behaviour is highly persistent in individuals with autism, with 77.8% of individuals continuing to show self-injury over three years (Richards et al. 2016). In summary, self-injury is common and persistent in autism, and the effects of self-injurious behaviour are highly detrimental to both the individual and their families. Thus, self-injury remains a behaviour of significant clinical importance in autism.

Despite the significant negative consequences of self-injurious behaviour, and the emerging evidence of persistence of the behaviour in autism, prevalence estimates for self-injurious behaviour in autism remain highly heterogeneous. Estimated prevalence rates vary from 33 to 71% (e.g. Baghdadli et al. 2003; Bartak and Rutter 1976; Bodfish et al. 2000; Cooper et al. 2009; Dominick et al. 2007; Gulsrud et al. 2018). This variation is likely due to a combination of differences in sample sizes and study methodologies, differences in the way that autism has been assessed and different definitions of self-injurious behaviour (Fombonne 2005). Moreover, participant characteristics such as age and presence and/or severity of co-morbid intellectual disability vary between studies making it difficult to estimate robustly the prevalence of self-injurious behaviour in autism. It is important that these differences are taken into account when attempting to synthesise prevalence data on self-injurious behaviour in autism in order to determine the extent to which these factors influence prevalence rates.

Our understanding of the factors that influence the presence and severity of self-injury within autism must be integrated with evidence-based models of the function and psychological mechanisms underpinning self-injury. For individuals with an intellectual disability, many of whom have co-morbid autism, the most influential and evidence-based explanatory model of self-injurious behaviour is derived from operant learning theory through an application of applied behaviour analysis (Summers et al. 2017). Within this model, self-injury is understood to occur as a result of positive or negative reinforcement and can be mediated by contingencies in the individual’s social, sensory and material environment. Evidence arising from applied behaviour analytic studies demonstrates that self-injury can be reduced by introducing communicative adaptive behaviours that displace self-injury, therefore providing evidence for self-injury as a functional behaviour (Oliver and Richards 2015). Consequently, interventions for behaviours that challenge are often predicated on the principles of applied behaviour analysis, such as those recommended in the NICE guidance on behaviours that challenge (NICE 2015).

However, there is preliminary emerging evidence that the presence of self-injury in those with autism without an intellectual disability may be best understood through reference to different psychological models than those applied to self-injury in those with co-morbid intellectual disability. Self-injury in those with autism without an intellectual disability may in fact be more similar to behaviours observed in those of typical development with mental health difficulties (Maddox et al. 2017). Often referred to as self-harm or non-suicidal self-injury (NSSI) in the general population, common topographies include cutting, carving and burning, and is often classified as distinct from the more repetitive and rhythmic self-injurious behaviours typically seen in those with an intellectual disability, such as head banging and self-hitting. Similarly to the distinction in the form of behaviour observed in those with mental health difficulties, the purpose of self-harm/NSSI within the autism population is believed to regulate negative affect and reduce emotional distress in those who engage in the behaviour (Maddox et al. 2017). Within this model, treatment techniques may more usefully draw on cognitive behavioural approaches, rather than applied behaviour analysis.

In light of this distinction between self-injurious behaviour in those with autism and self-injurious behaviour in those with autism and co-morbid intellectual disability, one initial step in furthering our understanding of this purported difference in categorisation is to evaluate the topographies of self-injurious behaviours in those with autism with and without intellectual disability; putative differences in underpinning psychological models may be associated with differences in form. For example, more rhythmic stereotypic topographies (e.g., head banging) may be associated with the presence of intellectual disability, whereas less rhythmic topographies (e.g., self-burning) may be associated with the *absence* of intellectual disability. More broadly, it is important to establish which features of autism and which demographic variables may influence the presence and/or topography of self-injurious behaviour in order to inform more accurate causal models in the future.

In summary, self-injurious behaviour is common, persistent and associated with poor outcomes. However, current prevalence estimates are highly varied and the influence of person and study characteristics on prevalence and topography of self-injury in autism is unknown. Thus, in order to identify existing service need for individuals with autism, and in turn shape future interventions and service delivery for this population, it is necessary to determine robust prevalence estimates for self-injurious behaviour. Similarly, there is a need to empirically synthesise differences in study and/or participant characteristics and quantitatively evaluate the effects of these factors on the data reporting self-injurious behaviour in those with autism. Therefore, the present meta-analysis seeks to describe and evaluate the current literature estimating the prevalence of self-injurious behaviour in autism in order to:

1. Synthesise prevalence rates of self-injurious behaviour in autism in order to generate robust estimates.
2. Evaluate how study characteristics (self-injurious behaviour definition, autism definition) and participant characteristics (age, gender, presence/severity of intellectual disability, presence/severity of autism) influence the reported prevalence rates.
3. Evaluate how participant characteristics influence the reported prevalence rates of individual topographies of self-injurious behaviour.

## Method

### Search Strategy

A broad list of search terms was derived from previous systematic reviews (Edmondson et al. 2015; Richards et al. 2015; Victor and Klonsky 2014). Literature searches were then conducted in Ovid PsychINFO, Ovid Medline and Ovid Embase by combining all variations of the autism and/or intellectual disability and self-injury search terms. The full list of autism and/or intellectual disability search terms were: Autism spectrum disorder*/autis* spectrum disorder*, Intellectual disabilit*, Autis*, ASD, PDD-NOS, PDDNOS, Unspecified PDD, Pervasive developmental disorder*, Pervasive developmental disorder not otherwise specified, Asperger* and Asperger* syndrome. The self-injury search terms were: Self-injur*, Self-injurious behav*, Self-injurious behaviour, Self-harm*, Self harm*, Selfharm*, Deliberate harm*, NSSI. Non-suicidal self-injur*, Nonsuicidal self-injur*, Self-mutilat*, Mutilat*, Self-cut*, Self-mutilative behav*, Deliberate self-harm*, DSH, Self-inflicted wound*, Self-destructive behav*, Self-destruct*, Parasuicid* and Non-fatal deliberate self-harm.

The literature search was conducted on 28th May 2018 and included all English language papers published from 1967 up to the date of the literature search. In addition to the literature search, a hand search of the reference lists of the returned articles was conducted and any relevant identified papers were included alongside those from the literature searches.

### Quality Review

Each article was reviewed based on five quality criteria (see Supplementary Materials 1 for table) which control for key threats to validity in sample selection (sample identification, assessment of autism, measurement of IQ, measurement of adaptive functioning and measurement of self-injurious behaviour). The quality criteria were adapted from Richards et al., (2015) and Surtees et al. (2018). For visual ease of interpretation, the criteria for each article were coded as red for a poor score of 0, yellow for an adequate score of 1, amber for a good score of 2 and green for an excellent score of 3. For example, for the criterion assessing the quality of measurement of self-injury/self-harm, a score of 0 (red) was attributed to papers that did not specify how self-injury was measured, a score of 1 (yellow) was attributed to papers that reported using non-validated informant report measures, a score of 2 (amber) was attributed to papers that used direct observation of self-injury *or* formal, validated informant report scales for self-injury and a score of 3 (green) was attributed to papers that obtained a consensus estimate of self-injury, combining data from multiple assessments including at least one direct observation *or* formal, validated scale.

This method provides a simple visual matrix that evidences the quality of each paper. The quality weighting for each paper was then calculated by dividing the total quality score by the maximum possible total of fifteen. All studies which met the inclusion criteria were reviewed by the author and rated for quality using these criteria. Inter-rater reliability was established by a second researcher independently reviewing a subset of studies (N = 8) to ascertain the reliability of the criteria. Inter-rater reliability for total quality weighting was good (*r* = 0.923; *p* = 0.001).

### Selection Strategy

A total of 3350 papers were identified by searching the databases. These papers were assessed for suitability for inclusion using the follow stages:

#### Stage 1: Screening

The titles and abstracts of all papers were screened by the researcher. Table 1 outlines the inclusion and exclusion criteria used to assess for suitability at this stage. In cases where suitability was unclear a second researcher reviewed the paper and consensus was derived.

**Table 1:** Inclusion and exclusion criteria for screening

Inclusion criteria	Exclusion criteria
Empirical papers	Conference proceedings, magazines, dissertations, review articles and books
Papers published are available in English	Papers published in a language other than English
Sample includes participants with idiopathic autism	Sample included participants with autism of known genetic cause, for example, fragile X Syndrome, tuberous sclerosis complex etc^a^
Abstract indicates that the paper reports on the prevalence and/or topography of self-injurious behaviour/self-harm within the autism group	Topography and/or prevalence of self-injurious behaviour/self-harm was not reported within the autism group
Cohort study	Case series and case studies

^a^Samples that included autism of known genetic cause such as fragile X Syndrome and tuberous sclerosis complex were excluded from this meta-analysis on the basis that it would not be possible to delineate self-injurious behaviour in autism from self-injurious behaviour associated with the genetic syndrome

#### Stage 2: Eligibility

The full texts of the screened papers were then read to assess the eligibility of the data for inclusion in the meta-analysis. The same inclusion and exclusion criteria were used at the screening and eligibility stages; however, the following additional criteria were applied to assess eligibility (see Table 2). A full list of the papers excluded at this stage are included in Supplementary Materials 2.

**Table 2:** Additional inclusion and exclusion criteria for eligibility assessment

Inclusion criteria	Exclusion criteria
Participants were recruited without any specific bias	Participants were recruited because they showed additional special characteristics that may influence self-injury e.g. epilepsy
Study reports on a unique sample (or a potentially overlapping sample, but the proportion of overlap cannot be readily determined)	Study reports on exactly the same sample as reported in a previous study

#### Stage 3: Quality Criteria

**Fig. 1:**
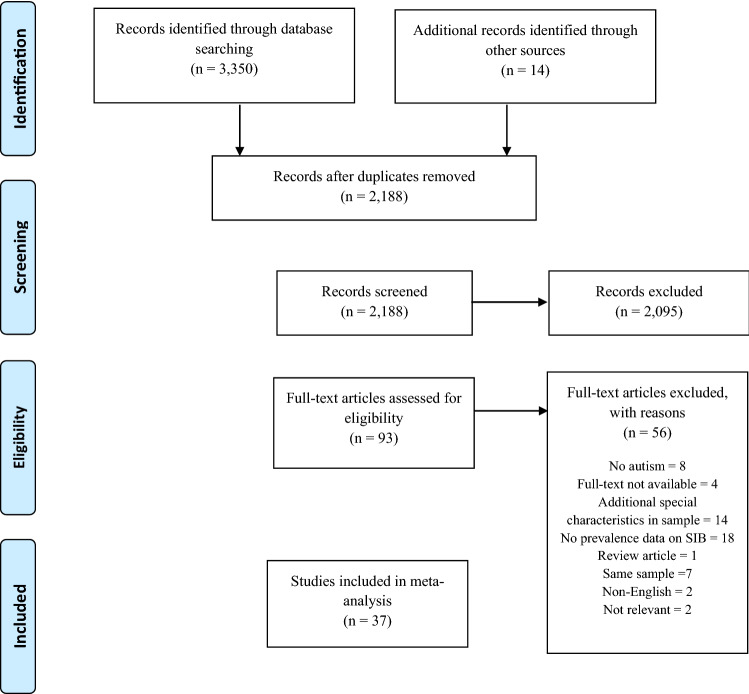
PRISMA flow chart showing the number of papers included and excluded at each stage of screening and review (Moher et al. 2009)

The quality of the remaining papers was then assessed according to the quality criteria outlined in Table 2. The flowchart presented below uses the PRISMA model (Moher et al. 2009) to outline the number of papers excluded at each stage of review (see Fig. 1).

### Data Analysis

In order to describe the prevalence of self-injurious behaviour in autism, the number of participants in the sample that displayed self-injurious behaviour^1^ was extracted from each paper. These data were analysed to generate pooled prevalence estimates. Given the substantial heterogeneity in the extracted prevalence rates between studies, a random-effects model used to calculate the pooled prevalence estimate. Fixed-effects models of pooled prevalence assume that that the true effect size for all studies under review are identical and that any variation in prevalence estimates are due to sampling error (Barendregt and Doi 2011). Given the substantial heterogeneity in the extracted prevalence rates between studies, a random-effects model was determined to be more appropriate and was used to calculate the pooled prevalence estimate. Unlike the fixed-effects model which attributes variation in prevalence rates to sampling error, the random-effects model assumes two sources of variability that could account for the heterogeneity between studies; one from sampling error and one from differences in study level characteristics, which are controlled for in the weighting assigned to each study. However, the random-effects model does not control for variability that arises due to differences in the methodological quality of the studies. Therefore, further sub-group analyses were also conducted in order to assess the impact of these differences upon heterogeneity.

**Table 3:**
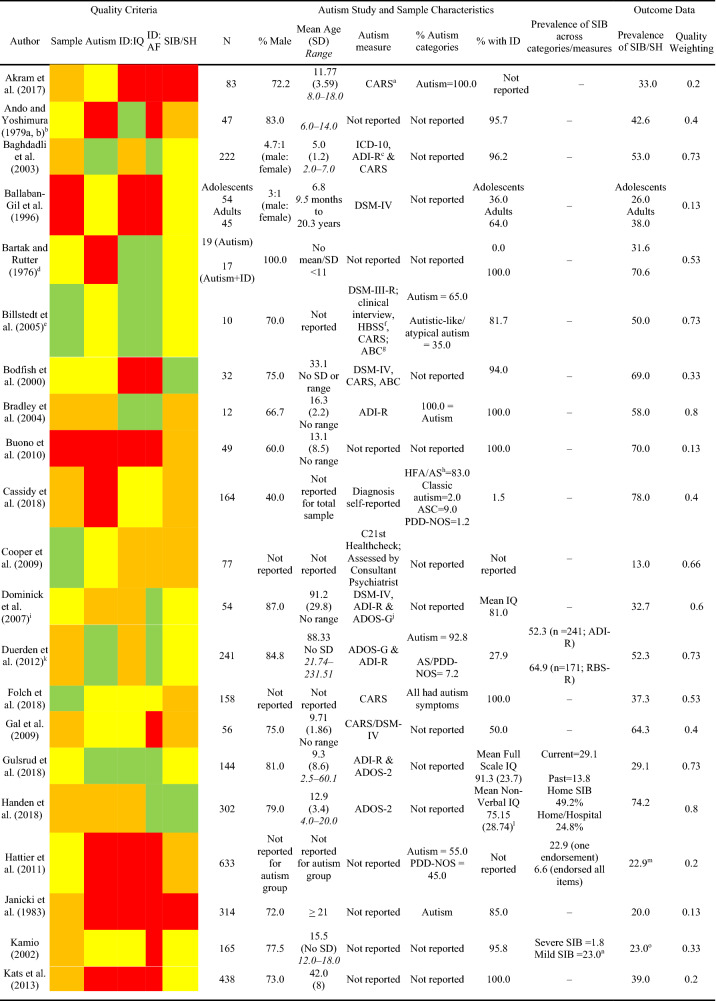

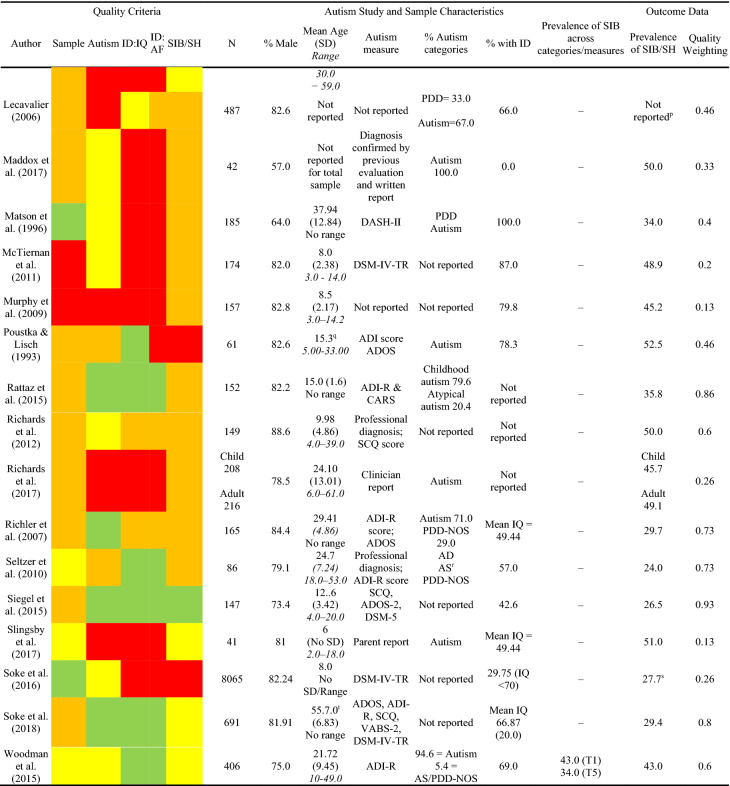
Quality criteria, study and sample characteristics and outcome data for studies reporting the prevalence/topography of SIB/SH in the autism population

In order to explore the influence of individual participant characteristics such as age, gender and presence of intellectual disability on prevalence rates of self-injurious behaviour, a series of meta-regression analyses were conducted. Due to insufficient data, it was not possible to meta-analyse the influence of autism severity on prevalence rates, however, those studies that did report these data are described.

In order to describe the prevalence rates of different topographies of self-injurious behaviour, the number of participants engaging in each topography was extracted from the papers that reported on topography. These data were analysed using the random-effects model to generate pooled prevalence estimates for each topography of behaviour.

Finally, in order to explore the impact of influential studies on the overall meta-analytic effect, a Baujat chart of sources of heterogeneity was used. Baujat et al. (2002) have proposed this method to explore heterogeneity which considers the impact of influential studies on the outcome of interest, in this instance, prevalence rates.

## Results

### Prevalence of Self-injury in Autism

A total of 37 primary studies reporting a total of 14,379 participants were identified as suitable for inclusion in the current meta-analysis. In order to assess the first aim of the meta-analysis, each study was evaluated against the quality criteria and data describing the study, sample characteristics (e.g. age, gender, autism characteristics, and presence of an intellectual disability) and total prevalence of self-injurious behaviour/self-harm were extracted and analysed to generate pooled prevalence estimates (see Table 3).

Across all studies, only five (13.5%) papers met criteria for the highest quality rating for sample identification, seven (18.9%) for autism assessment, 11 (29.7%) for assessment of IQ, 13 (35.1%) for assessment of adaptive functioning and three (8.1%) for measurement of self-injurious behaviour. Whilst the majority of papers (N = 25; 67.5%) reported the proportion of the sample that had an intellectual disability, assessment of intellectual disability received the highest number of poor ratings with 14 (37%) studies for assessment of IQ and 18 studies (48.6%) for assessment of adaptive functioning being rated as poor. In addition to reporting the prevalence of self-injurious behaviour, 16 studies (43.2%) reported prevalence of different topographies of behaviour. No studies were excluded on the basis of quality as doing so would result in the loss of valuable data obtained from large samples of participants. Instead, analyses of the effect of methodological quality was conducted to determine the possible influence of poorer quality studies on prevalence estimates. Just over 50% of studies received a good (N = 19; 51.3%) rating for sample identification suggesting that the prevalence data reported in these studies were obtained from representative samples.

**Fig. 2:**
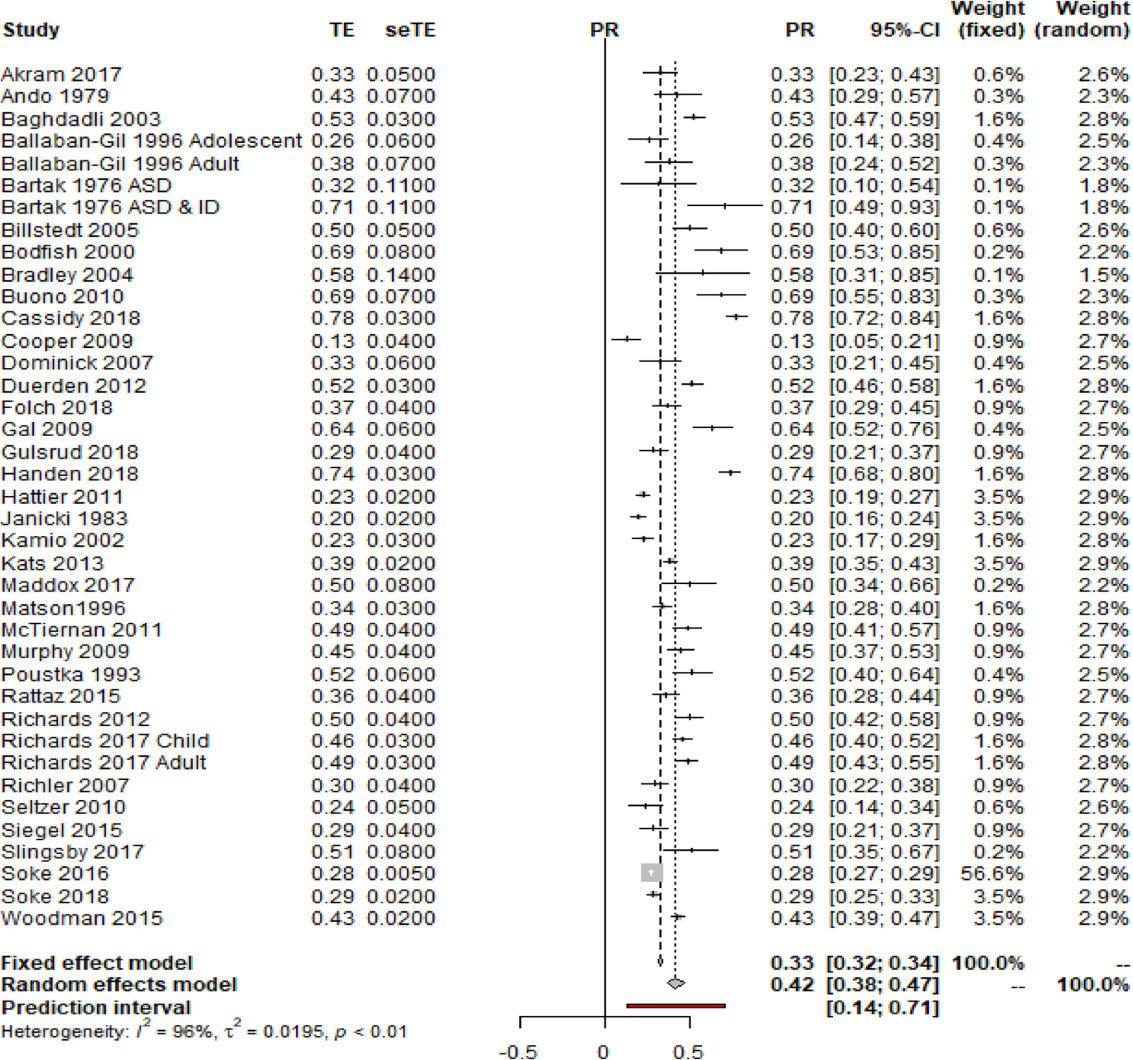
Pooled prevalence estimates for self-injurious behaviour in autism using a random-effects model. Treatment effect (TE), standard error of the treatment effect (seTE), prevalence rate, confidence intervals and weighting by the random-effects model are reported

In order to achieve the first aim of the meta-analysis, prevalence rates of self-injurious behaviour in autism were synthesised and a random-effects pooled prevalence estimate was generated based on the 37 studies that met the inclusion criteria (see Fig. 2).

The random-effects model was calculated using the generic inverse variance method and generated a weighted prevalence estimate of 42% (z = 17.71, p ≤ 0.001; CI 38–47%) for self-injurious behaviour in autism.

An unacceptable level of heterogeneity between the prevalence rates reported in the primary studies was observed (tau^2^ = 0.019, Higgin’s I^2^ = 96%; Q = 958.84, p < 0.001), which suggests that the estimates of the primary studies are biased by the presence of uncontrolled and potentially confounding factors.

### Heterogeneity due to Study Level Characteristics

In order to assess the impact of study level characteristics upon heterogeneity, a series of subgroup analyses were conducted on the prevalence rates of self-injurious behaviour based on the quality ratings of poor, adequate, good and excellent for each of the five types of methodological bias (see Table 4).

**Table 4:** Sub-group analysis of the prevalence rates (%) of SIB based on the quality ratings for each type of methodological bias

	Poor	Adequate	Good	Excellent	Q	*p*
Sample identification	45.0	40.0	45.0	32.0	6.24	0.10
Assessment of autism	46.0	39.0	47.0	36.0	2.32	0.50
Measurement of ID: IQ	40.0	50.0	43.0	39.0	0.90	0.82
Measurement of IQ: adaptive	41.0	57.0	31.0	43.0	1.74	0.62
Measurement of SIB/SH	31.0	39.0	43.0	57.0	6.48	0.09

A mixed effects ANOVA was computed to test the difference between the proportions (Q) with associated probability levels (*p*)

The results of the sub-group analysis indicated that none of the indices of risk of bias evidenced significant differences in the estimated prevalence of self-injurious behaviour.

### Impact of Influential Studies

**Fig. 3:**
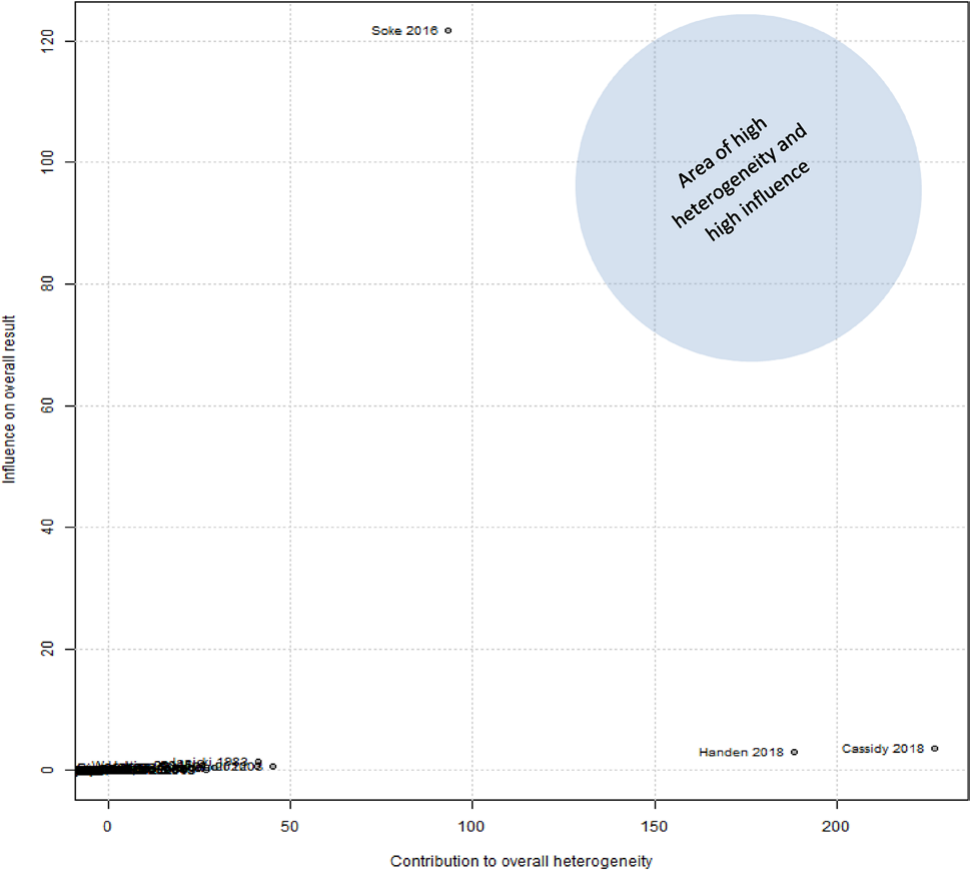
Baujat chart of sources of heterogeneity

To examine the influence of individual studies to the overall effect, a baujat chart of sources of heterogeneity was plotted (see Fig. 3). On the x-axis the contribution of each study to the overall heterogeneity statistic is plotted; higher values on the x-axis reflect increasing heterogeneity associated with the omission of a study. On the y-axis the standardised difference of the overall treatment effect with and without each study is plotted; this quantity describes the influence of each study on the overall effect and higher values on the y-axis reflect greater change in the omnibus effect associated with the omission of a study. No studies fell within the area of high heterogeneity and high influence, although the study by Soke et al., (2016) exerted the highest influence on the overall effect.

**Fig. 4:**
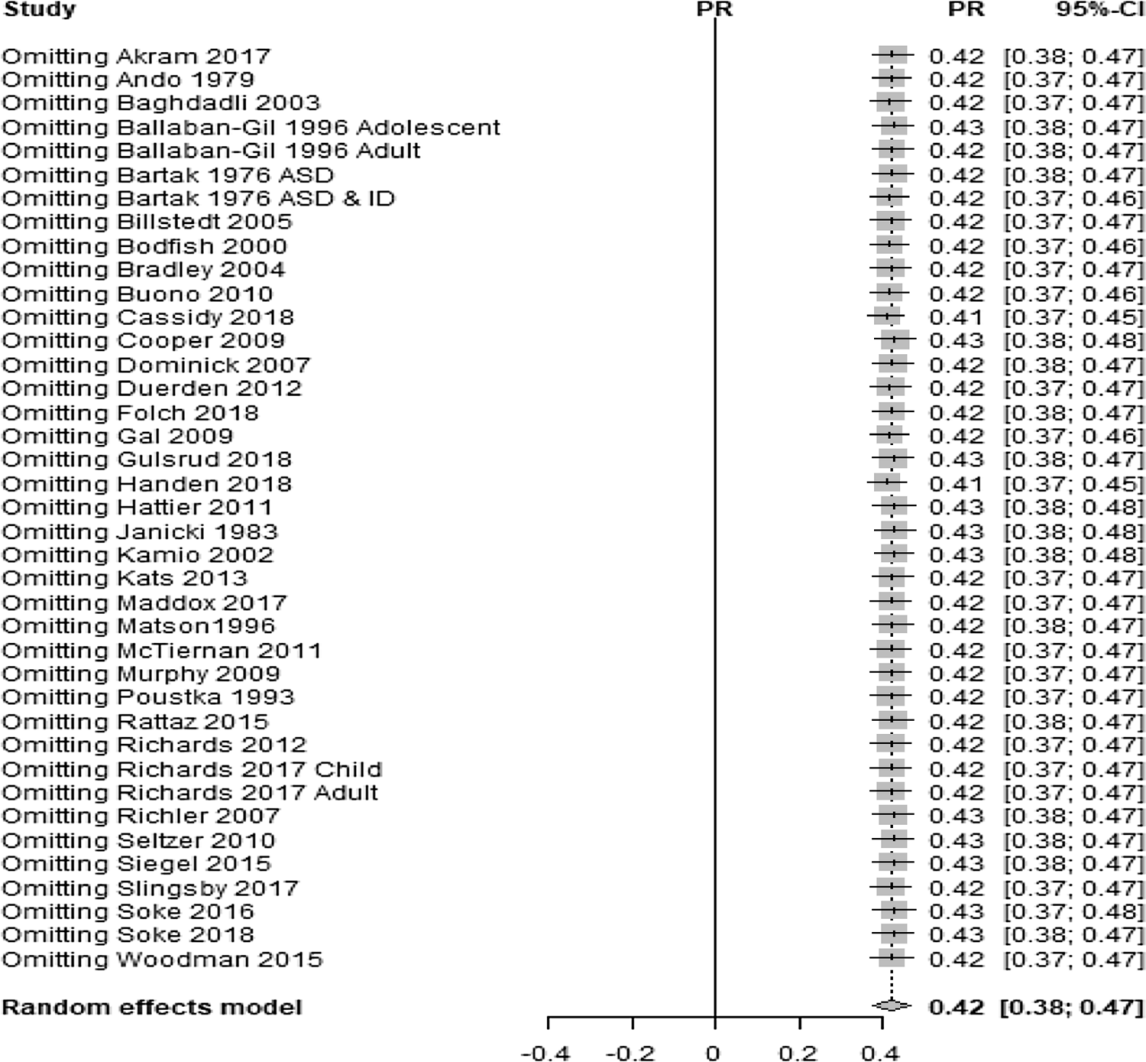
Pooled prevalence estimate for self-injurious behaviour in autism using a random-effects model with each study omitted. Prevalence rates and confidence intervals are reported

Second, to examine whether any particular study or studies exerted a disproportionately high influence on the overall meta-analytic effect, a “leave-one-out” analysis, in which the random effects model was calculated with each of the primary studies removed in turn, was conducted (see Fig. 4). If omitting a study results in an effect that lies outside of the 95% CI for the complete meta-analysis then that study is deemed to have a disproportionate influence and is removed from the omnibus test. The study by Soke et al. (2016) strongly influenced the overall result but was not markedly heterogeneous to the other studies. The reason why it exerted influence over the overall result was due to sample size (N = 8065) and therefore this study was not considered to be problematic.

The “leave-one-out” analysis did not alter the prevalence estimate, including the very large sample in the Soke et al. (2016) paper, and therefore no studies were considered disproportionally influential on the overall meta-analytic effect.

### Influence of Participant Characteristics on Prevalence Rates

In order to explore the second aim of the meta-analysis, the influence of age, gender and presence of an intellectual disability on prevalence rates was assessed using meta-regression analysis (see Table 5). Mean age of participants was extracted from 25 (67.5%) studies, percentage of male participants from 29 (78.3%) studies and percentage of participants with an intellectual disability from 27 (72.9%) studies.

**Table 5:** Participant characteristic influencing prevalence of SIB in autism

Covariate	Estimate	S.E.	Z	*p*	Lower 95%CI	Upper 95%CI
Age (mean age)	− 0.0028	0.0024	− 1.174	.240	− 0.0074	0.0019
Gender (% male)	− 0.0063	0.0025	− 2.469	.013*	− 0.0113	− 0.0013
Presence of ID	− 0.0001	0.0010	− 0.048	.961	− 0.0021	0.0020

The meta-regression analysis in Table 5 revealed that the association between mean age of participants and prevalence rates of self-injury, and the proportion of participants with an intellectual disability and prevalence rates of self-injury were non-significant (p = 0.240) and (p = 0.961), respectively. However, the meta-regression analysis revealed a significant association between the proportion of male participants and prevalence rates (*p* = 0.013) suggesting that gender may influence the prevalence of self-injurious behaviour in autism. As the percentage of males included in the sample increased, the prevalence of self-injury significantly decreased. Specifically, for every unit increase in the percentage of males in the sample, prevalence of SIB decreased by 0.0063%.

Due to insufficient data it was not possible to meta-analyse the effect of severity of autism on prevalence of self-injurious behaviour. Only two studies reported prevalence of self-injury across different levels of autism. These studies found that severity of autism is associated with a higher prevalence of self-injury, and moreover, that severity of self-injurious behaviour is associated with more severe autism (Akram et al. 2017; Folch et al. 2018).

Additionally, it was not possible to meta-analyse the prevalence of self-injurious behaviour across levels of intellectual disability due to insufficient data, however, one study reported prevalence data in this way. Ballaban-Gil et al. (1996) reported the prevalence of self-injurious behaviour within those with normal/near normal cognitive ability, mild-moderate intellectual disability and severe intellectual disability in an adult and adolescent sample. Within the adult sample, self-injury was displayed by 31% of participants with normal/near normal cognitive ability, 9% with mild-moderate intellectual disability and 32% with severe intellectual disability. Within the adolescent group, self-injury was displayed by 9% of participants with normal/near normal cognitive ability, 45% with mild-moderate intellectual disability and 30% with severe intellectual disability. Within both samples, those with severe intellectual disability showed similar prevalence rates of self-injury (32% and 30%, respectively). However, rates varied considerably between those with a mild-moderate intellectual disability. Forty-five percent of participants in the adolescent sample displayed self-injurious behaviour compared to 9% in the adult sample.

### Prevalence of Different Topographies of Self-injurious Behaviour

A total of 14 studies reported on the prevalence of different topographies of self-injurious behaviour (N = 21). In order to address the final aim of the meta-analysis, these prevalence data were extracted and analysed to generate pooled prevalence estimates. Each topography of behaviour was included in the meta-analysis if a minimum of two studies reported data for the behaviour (see Table 6).

**Table 6:**
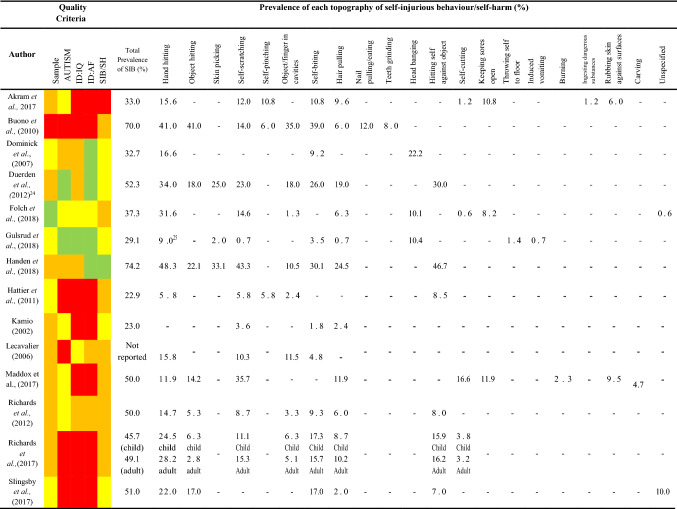
Quality criteria, total prevalence of self-injurious behaviour in the total sample and prevalence of topographies of SIB/SH in the autism population

**Fig. 5:**
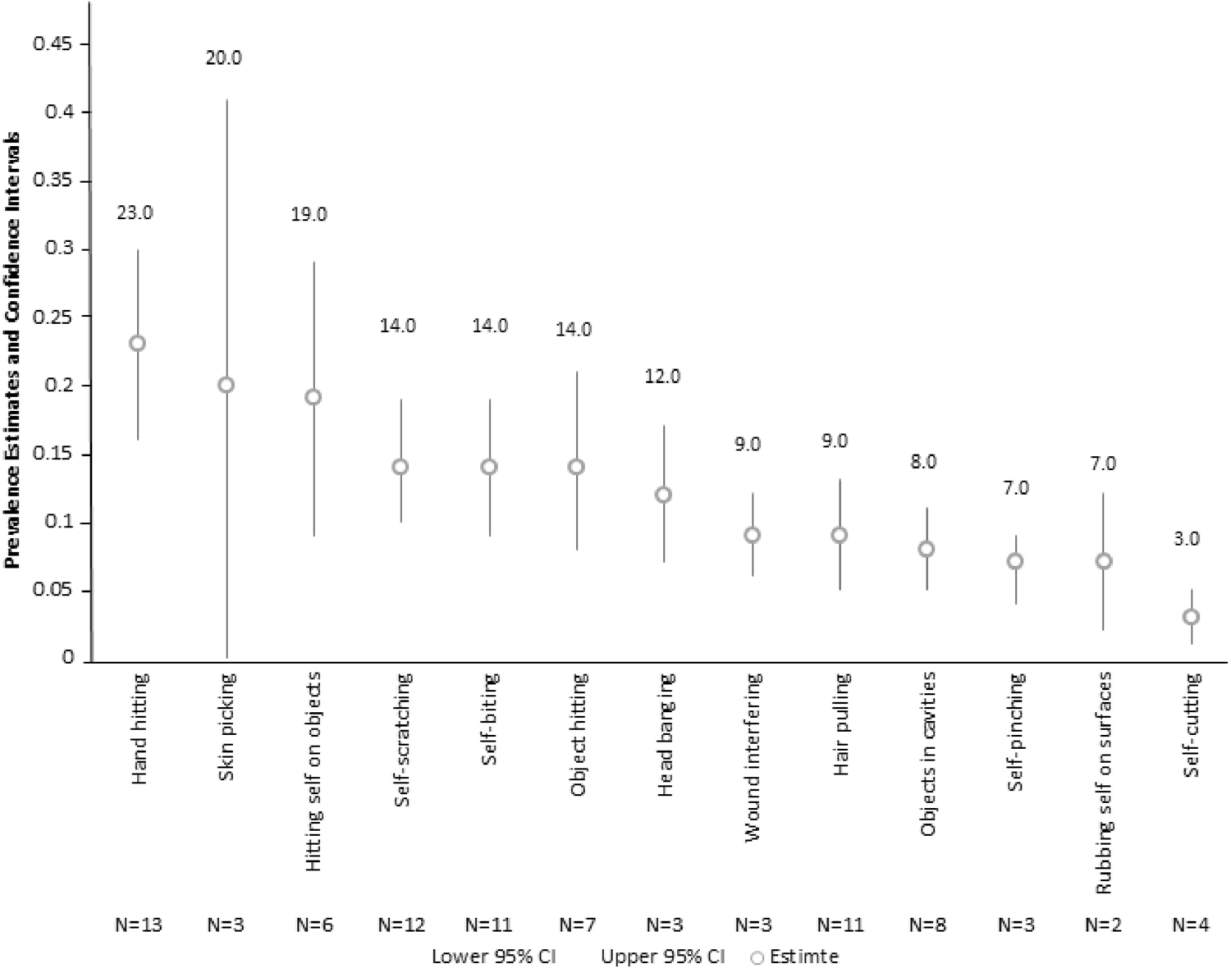
Pooled prevalence estimates for self-injurious behaviour in autism using a random-effects model. Treatment effect (TE), standard error of the treatment effect (seTE), prevalence rate, confidence intervals and weighting by the random-effects model are reported

A total of 13 topographies of self-injurious behaviour were analysed. Topographies that were only reported by one study were excluded from the meta-analysis (N = 8). For brevity, the forest plots, prevalence rates, confidence intervals and weighting assigned by the random effects model for each topography of behaviour can be found in Supplementary Materials 3. For studies that calculated prevalence of topography as a percentage of the number that showed self-injury, this was converted to a percentage of the total sample in order to generate pooled prevalence estimates. Figure 5 presents the pooled random effects prevalence with associated confidence intervals for each of the 13 topographies.

### Influence of Participant Characteristics on Prevalence Rates of Different Topographies of Self-injurious Behaviour

In order to conduct a meta-regression analysis, it is recommended to include at least ten studies which report data on the outcome of interest (Higgins 2008). As such, meta-regression analyses looking at the influence of participant characteristics on prevalence of behaviour topography were only conducted on four topographies that had sufficient studies reporting data; hair pulling, hand hitting, self-biting and self-scratching. However, not all of the papers that reported data on these topographies included data on intellectual disability, age and gender, therefore, the results of the meta-regression analysis should be interpreted with caution (Table 7).

**Table 7:** Participant characteristics influencing prevalence of hair pulling, hand biting, self-biting and self-scratching

	Covariate	Estimate	S.E.	Z	*p*	Lower 95%CI	Upper 95%CI
Hair pulling	Age (mean age)	0.0014	0.0116	0.123	.901	− 0.0212	0.0241
	Gender (% male)	< 0.0001	0.0029	0.008	.993	0.0057	0.0057
	Presence of ID	− 0.0013	0.0005	− 2.616	.008*	− 0.0023	− 0.0003
Hand hitting	Age (mean age)	0.0245	0.022	1.111	.266	− 0.0187	0.0676
	Gender (% male)	− 0.0014	0.0045	− 0.305	.760	− 0.0101	0.0074
	Presence of ID	0.0013	0.0013	1.020	.307	− 0.0012	0.0038
Self-biting	Age (mean age)	0.0006	0.0138	0.042	.966	− 0.0264	0.0275
	Gender (% male)	− 0.0072	0.0043	− 1.695	.09	− 0.0155	0.0011
	Presence of ID	− 0.0011	0.0016	− 0.667	.504	− 0.0043	0.0021
Self-scratching	Age (mean age)	0	0.0236	0	.585	− 0.3902	0.6907
	Gender (% male)	− 0.004	0.0036	− 1.130	.269	− 0.0111	0.0031
	Presence of ID	− 0.0021	0.0007	− 3.025	.002*	− 0.0035	− 0.0008

The association between the proportion of participants with an intellectual disability and prevalence of hair pulling and self-scratching was found to be significant (*p* = 0.008 and *p* = 0.002, respectively) suggesting that presence of an intellectual disability may increase the prevalence of these topographies of self-injurious behaviour. However, there were no other significant associations between participant characteristics and prevalence of different topographies of self-injurious behaviour.

## Discussion

The prevalence of self-injurious behaviour in autism was meta-analysed in this study to generate a robust pooled prevalence estimate. Sub-group analyses were completed in order to evaluate how study and participant characteristics influenced the overall reported prevalence rates of self-injury, as well as the prevalence of individual topographies of behaviour. This is the first meta-analysis to synthesise prevalence rates of self-injurious behaviour in autism, and thus is important both clinically and scientifically. The meta-analysis employed a thorough, robust and careful search strategy, adopting key search terms from published literature within the field. Moreover, the use of robust quality criteria further strengthens the findings in this meta-analysis. In summary, prevalence rates of self-injurious behaviour extracted from 37 primary studies were analysed in this meta-analysis, generating a pooled prevalence estimate of 42% for self-injurious behaviour in autism. There were no significant associations between mean age and proportion of those with an intellectual disability and prevalence of self-injury, however, gender did have an effect on prevalence rates with a higher proportion of male participants associated with a slight decrease in prevalence of self-injurious behaviour. Analysis of the topography data showed hand hitting, skin picking and hitting self against objects to be the most common forms of self-injury, whilst self-cutting was the least common.

The overall pooled prevalence estimate for self-injury in autism is 42%, which is significantly higher than prevalence estimates for self-harm in the typically developing population. For example, the prevalence of self-harm in typically developing children and adolescents is approximately 8% and 5.9% in adults (Klonsky 2011; Morgan et al. 2017). The difference between these prevalence rates are striking and suggests that those with autism are a particularly high risk group for self-injury.

A substantial amount of heterogeneity between studies included in the prevalence estimate was observed (I^2^ = 96%), therefore it was necessary to conduct further analyses to explore the factors which may have been contributing to the level of heterogeneity. Sub-group analyses revealed that differences in the methodologies and quality of the primary studies did not influence prevalence rates. The influence of participant characteristics such as age, gender and presence of an intellectual disability on reported prevalence rates were analysed using meta-regression. Interestingly, gender was found to be significantly associated with prevalence of self-injurious behaviour with greater number of males in the sample associated with a slight decrease in prevalence rates. Previous research has replicated these results demonstrating an association between self-injurious behaviour and female gender (Cohen et al. 2010). Similarly, in a study exploring characteristics of females and males with autism, Frazier et al. (2014) found that females exhibited more externalizing behaviours, such as self-injury, compared to males. However, in a sample of individuals with autism, Baghdadli et al. (2003) found no association between self-injury and gender. Therefore, further research may be warranted in order to replicate and confirm the association between self-injury and female gender. Given the heightened prevalence of autism diagnosis in males than in females, the association between self-injury and gender is of particular importance (Saracino et al. 2010). Females are commonly underrepresented in autism research due to the inherent difficulties in identifying and diagnosing autism within this group. Particularly in females with intellectual disability, their presentation of symptoms may be misinterpreted and accurate diagnosis is often delayed (Halladay et al. 2015). Therefore, in order to explore the association between gender and self-injury further, future research should seek to include more female participants in their studies.

There were no significant associations between age and prevalence of self-injury. Within the literature, the reported associations between age and self-injury in autism vary. In their study, Esbensen et al. (2009) reported a correlation between older age and significantly lower levels of self-injury, whilst Baghdadli et al. (2003) found no significant association between younger age and self-injury. In a sample of individuals with an intellectual disability of heterogeneous aetiology, Oliver et al. (1987) demonstrated a curvilinear relationship between age and self-injury, with self-injury peaking between the ages of 15 and 25. The authors suggest that the increase in prevalence of self-injury can be explained by the operant model which suggests that self-injury becomes learnt and is shaped by the environment. Given the high prevalence of self-injury in autism, there is a need for further research exploring the associations between age and self-injury. The majority of studies included in this meta-analysis recruited participants under the age of 18 (72%), therefore, there is a clear need for future studies to include participant samples that contain individuals with autism from across the lifespan. Furthermore, there is a need for longitudinal studies to evaluate changes in self-injury over time and across the lifespan.

There was no significant association between presence of an intellectual disability and prevalence of self-injury. Previous research has identified the presence of an intellectual disability as the most common risk marker for self-injurious behaviour (McClintock et al. 2003), therefore, the lack of association between prevalence of self-injury and intellectual disability was unexpected. It might be that in autism, the absence of an intellectual disability is not as protective against self-injury or self-harm as might be assumed. Given the emerging evidence of the distinction between self-injury in autism and self-injury in autism with co-morbid intellectual disability, it might be that presence of an intellectual disability influences the form and function of self-injury rather than the prevalence of the behaviour. Recent research has suggested that self-injury in those with autism without an intellectual disability may be more similar to behaviours observed in those of typical development with mental health difficulties and may serve a similar function (Maddox et al. 2017). The results of the present meta-analysis have not supported a distinction between self-injury and self-harm in autistic people with and without an intellectual disability respectively. Significant further research regarding the form and function and self-injury and self-harm is warranted to inform more accurate causal models. Furthermore, the results from studies such as Maddox et al. (2017) may suggest a need for services that are tailored towards individuals with autism without intellectual disability who present with self-injurious behaviour, particularly given the link between self-harm and suicide and the high rates of death by suicide in the autism population (Cassidy et al. 2014; Kirby et al. 2019; Richa et al. 2014). However, the overall quality of assessment of intellectual disability across studies was poor. It is therefore possible that the lack of association between intellectual disability and prevalence of self-injurious behaviour is related to poor assessment of intellectual disability, therefore, these findings should be interpreted cautiously.

Finally, in order to explore the influence of participant characteristics on prevalence of different topographies of self-injury, meta-regression analyses were conducted on the available data. The results of the analysis highlighted an association between the proportion of participants with an intellectual disability and prevalence of hair pulling and self-scratching, suggesting that intellectual disability may influence these topographies of behaviour. However, due to the limited number of data points available for inclusion in the analysis, these results should be interpreted with caution. Overall, few studies reported on the prevalence of different topographies of behaviour making it difficult to explore any differences between those with autism only and those with autism *and* co-morbid intellectual disability. Given the emerging evidence that self-injury in those with autism may serve a different function to that observed in those with autism and co-morbid intellectual disability, there is a growing need for research to focus on behavioural topography within these two groups. Specifically, increasing our understanding of the differences in the form and function of self-injury in these two clinical groups is of particular importance for shaping interventions. For example, management of self-injury in individuals with autism and no intellectual disability might be better suited to self-harm interventions typically offered to people with mental health difficulties, such as cognitive behavioural therapy (CBT) and dialectical behaviour therapy (DBT), rather than interventions solely derived from applied behaviour analysis.

Whilst the meta-analysis was robust and inclusive, the study did have some limitations. Due to insufficient data, it was not possible to analyse prevalence of self-injury across different levels of autism severity. However, those studies that did report data on this suggests that greater severity of autism is associated with increased prevalence of self-injury. In order to further our understanding of the influence of autism severity on prevalence of self-injurious behaviour, autism symptomology should be operationalised more clearly and reported in future studies. Secondly, the meta-analysis was not pre-registered. Future meta-analyses in this area should seek to pre-register the study protocol to improve replicability and reduce bias.

In summary, the meta-analysis has generated a robust estimate of 42% for the prevalence of self-injurious behaviour in autism. Despite significant heterogeneity in the studies, the prevalence of self-injurious behaviour was not affected. The analysis found no association between intellectual disability, age and self-injury. However, gender was found to be significantly associated with self-injury, with more males in the sample being associated with a slightly lower prevalence of self-injury. Analysis of the topography data showed hand hitting, skin picking and hitting self against objects to be the most common forms of self-injury, whilst self-cutting was the least common.

## Electronic supplementary material

Below is the link to the electronic supplementary material.

Supplementary file1 (PDF 13 kb)

Supplementary file2 (PDF 152 kb)

Supplementary file3 (PDF 893 kb)

## Footnotes

1 Where a study reports more than one prevalence figure for self-injurious behaviour, a decision on which figure to include in the meta-analysis was made on an individual basis. Reasons for the decision are included as a footnote for each study in Table 3.

## References

[CR1] Akram B, Batool M, Rafi Z, Akram A (2017). Prevalence and predictors of non-suicidal self-injury among children with autism spectrum disorder. Pakistan Journal of Medical Sciences.

[CR2] Allen D, Lowe K, Brophy S, Moore K (2009). Predictors of restrictive reactive strategy use in people with challenging behaviour. Journal of Applied Research in Intellectual Disabilities.

[CR3] Altiere MJ, von Kluge S (2009). Family functioning and coping behaviors in parents of children with autism. Journal of Child and Family Studies.

[CR4] Ando H, Yoshimura I (1979). Speech skill levels and prevalence of maladaptive behaviors in autistic and mentally retarded children. Child Psychiatry and Human Development.

[CR5] Ando H, Yoshimura I (1979). Comprehension skill levels and prevalence of maladaptive behaviors in autistic and mentally retarded children. Child Psychiatry and Human Development.

[CR6] Baghdadli A, Pascal C, Grisi S, Aussilloux C (2003). Risk factors for self-injurious behaviours among 222 young children with autistic disorders. Journal of Intellectual Disability Research.

[CR7] Baghdadli A, Pry R, Michelon C, Rattaz C (2014). Impact of autism in adolescents on parental quality of life. Quality of Life Research.

[CR8] Baird G, Simonoff E, Pickles A, Chandler S, Loucas T, Meldrum D (2006). Prevalence of disorders of the autism spectrum in a population cohort of children in South Thames: The Special Needs and Autism Project (SNAP). Lancet.

[CR9] Ballaban-Gil K, Rapin I, Tuchman R, Shinnar S (1996). Longitudinal examination of the behavioral, language, and social changes in a population of adolescents and young adults with autistic disorder. Pediatric Neurology.

[CR100] Barendregt, J. J., & Doi, S. A. (2011). Meta XL User Guide. Version 2.0.

[CR10] Bartak L, Rutter M (1976). Difference between mentally retarded and normally intelligent autistic children. Journal of Autism and Childhood Schizophrenia.

[CR11] Baujat B, Mahé C, Pignon JP, Hill C (2002). A graphical method for exploring heterogeneity in meta-analysis: Application to a meta-analysis of 65 trials. Statistics in Medicine.

[CR12] Beadle-Brown J, Murphy G, DiTerlizzi M (2009). Quality of life for the Camberwell Cohort. Journal of Applied Research in Intellectual Disabilities.

[CR13] Billstedt E, Gillberg C, Gillberg C (2005). Autism after adolescence: Population-based 13- to 22-year follow up study of 120 individuals with autism diagnosed in childhood. Journal of Autism and Developmental Disorders.

[CR14] Bodfish JW, Symons FJ, Parker DE, Lewis MH (2000). Varieties of repetitive behavior in autism: Comparisons to mental retardation. Journal of Autism and Developmental Disorders.

[CR15] Bradley EA, Summers JA, Wood HL, Bryson SE (2004). Comparing rates of psychiatric and behavior disorder in adolescents and young adults with severe intellectual disability with and without autism. Journal of Autism and Developmental Disorders.

[CR16] Brereton AV, Tonge BJ, Einfeld SL (2006). Psychopathology in children and adolescents with autism compared to young people with intellectual disability. Journal of Autism and Developmental Disorders.

[CR17] Buono S, Scanella P, Palmigiano M (2010). Self-injurious behavior: A comparison between Prader-Willi Syndrome, Down Syndrome and Autism. Life Span and Disability.

[CR18] Cassidy S, Bradley P, Robinson J, Allison C, McHugh M, Baron-Cohen S (2014). Suicidal ideation and suicide plans or attempts in adults with Asperger’s syndrome attending a specialist diagnostic clinic: A clinical cohort study. The Lancet Psychiatry.

[CR19] Cassidy S, Bradley L, Shaw R, Baron-Cohen S (2018). Risk markers for suicidality in autistic adults. Molecular Autism.

[CR20] Center for Disease Control (2009). Prevalence of autism spectrum disorders Autism and Developmental Disabilities Monitoring Network, United States, 2006. MMWR Surveillance.

[CR21] Center for Disease Control (2014). Prevalence of autism spectrum disorders—Autism and Developmental Disabilities Monitoring Network, United States, 2010. MMWR Surveillance.

[CR22] Cohen I, Tsiouris J, Flory M, Kim SY, Freedland R, Heaney G (2010). A large scale study of the psychometric characteristics of the IBR modified overt aggression scale: Findings and evidence for increased self-destructive behaviors in adult females with autism spectrum disorder. Journal of Autism and Developmental Disorders.

[CR23] Cooper SA, Smiley E, Allan LM, Jackson A, Finlayson J, Mantry D (2009). Adults with intellectual disabilities: Prevalence, incidence and remission of self-injurious behaviour, and related factors. Journal of Intellectual Disability Research.

[CR24] Davies L, Oliver C (2013). The age related prevalence of aggression and self-injury in persons with an intellectual disability: A review. Research in Developmental Disabilities.

[CR25] Dominick KC, Davis NO, Lainhart J, Tager-Flusberg H, Folstein S (2007). Atypical behaviors in children with autism and children with a history of language impairment. Research in Developmental Disabilities.

[CR26] Duerden E, Oakley H, Mak-Fan K, McGrath P, Taylor M (2012). Risk factors associated with self-injurious behaviors in children and adolescents with autism spectrum disorders. Journal of Autism and Developmental Disorders.

[CR27] Edmondson AJ, Brennan CA, House AO (2015). Non-suicidal reasons for self-harm. A systematic review of self-reported accounts. Journal of Affective Disorders.

[CR28] Esbensen AJ, Greenberg JS, Seltzer MM, Aman MG (2009). A longitudinal investigation of psychotropic and non-psychotropic medication use among adolescents and adults with autism spectrum disorders. Journal of Autism and Developmental Disorders.

[CR29] Folch A, Cortes MJ, Salvador-Carulla L, Vicens P, Irazabal M, Munoz S (2018). Risk factors and topographies for self-injurious behaviour in a sample of adults with intellectual developmental disorders. Journal of Intellectual Disability Research.

[CR30] Fombonne E (2005). Epidemiology of autistic disorder and other pervasive developmental disorders. Journal of Clinical Psychiatry.

[CR31] Fombonne E (2009). Epidemiology of pervasive developmental disorders. Pediatric Research.

[CR32] Frazier TW, Georgiades S, Bishop SL, Hardan AY (2014). Behavioral and cognitive characteristics of females and males with autism in the Simons Simplex collection. Journal of the American Academy of Child and Adolescent Psychiatry.

[CR33] Gal E, Dyck M, Passmore A (2009). The relationship between stereotyped movements and self-injurious behavior in children with developmental or sensory disabilities. Research in Developmental Disabilities.

[CR34] Ghaziuddin M, Tsai L, Ghaziuddin N (1992). Comorbidity of autistic disorder in children and adolescents. European Child Adolescent Psychiatry.

[CR35] Greenberg JS, Seltzer MM, Hong J, Orsmond GI (2006). Bidirectional effects of expressed emotion and behavior problems and symptoms in adolescents and adults with autism. American Journal on Mental Retardation.

[CR36] Gulsrud A, Lin CE, Park MN, Hellemann G, McCracken J (2018). Self-injurious behaviours in children and adults with autism spectrum disorder (ASD). Journal of Intellectual Disability Research.

[CR37] Halladay AK, Bishop S, Constantino JN, Daniels AM, Koenig K, Palmer K (2015). Sex and gender differences in autism spectrum disorder: Summarizing evidence gaps and identifying emerging areas of priority. Molecular Autism.

[CR38] Handen BL, Mazefsky CA, Gabriels RL, Pedersen KA, Wallace M, Siegel M (2018). Risk factors for self-injurious behavior in an inpatient psychiatric sample of children with autism spectrum disorder: A naturalistic observation study. Journal of Autism and Developmental Disorders.

[CR40] Hastings RP (2003). Child behaviour problems and partner mental health as correlate of stress in mothers and fathers of children with autism. Journal of Intellectual Disability Research.

[CR39] Hastings RP, Brown T (2002). Coping strategies and the impact of challenging behaviours on special educators burnout. Mental Retardation.

[CR41] Hattier MA, Matson JL, Belva BC, Horovitz M (2011). The occurrence of challenging behaviours in children with autism spectrum disorders and atypical development. Developmental Neurorehabilitation.

[CR42] Higgins JP, Green S (2008). Cochrane handbook for systematic reviews of interventions.

[CR43] Holden B, Gitlesen JP (2006). A total population study of challenging behaviour in the county of Hedmark, Norway: Prevalence, and risk markers. Research in Developmental Disabilities.

[CR44] Iwata BA, Dorsey MF, Slifer KJ, Bauman KE, Richman GS (1994). Toward a functional-analysis of self-injury. Analysis and Intervention in Developmental Disabilities.

[CR45] Janicki MP, Jacobson JW (1983). Selected clinical-features and service characteristics of autistic adults. Psychological Reports.

[CR46] Kalb LG, Stuart EA, Freedman B, Zablotsky B, Vasa R (2012). Psychiatric-related emergency department visits among children with an autism spectrum disorder. Pediatric Emergency Care.

[CR47] Kamio Y (2002). Self-injurious and aggressive behaviour in adolescents with intellectual disabilities: A comparison of adolescents with and without autism. Japanese Journal of Special Education..

[CR48] Kats D, Payne L, Parlier M, Piven J (2013). Prevalence of selected clinical problems in older adults with autism and intellectual disability. Journal of Neurodevelopmental Disorders.

[CR49] Kirby AV, Bakian AV, Zhang Y, Bilder DA, Keeshin BR, Coon H (2019). A 20-year study of suicide death in a statewide autism population. Autism Research.

[CR50] Klonsky ED (2011). Non-suicidal self-injury in United States adults: Prevalence, sociodemographics, topography and functions. Psychological Medicine.

[CR51] Knapp M, Comas-Herrera A, Astin J, Beecham J, Pendaries C (2005). Intellectual disability, challenging behaviour and cost in care accommodation: what are the links?. Health & Social Care in the Community.

[CR52] Knapp M, Romeo R, Beecham J (2009). Economic cost of autism in the UK. Autism.

[CR53] Lecavalier L (2006). Behavioural and emotional problems in young people with Pervasive Developmental Disorders: Relative prevalence, effects of subject charachteristics and empirical classification. Journal of Autism and Developmental Disorders.

[CR54] Lecavalier L, Leone S, Wiltz J (2006). The impact of behaviour problems on caregiver stress in young people with autism spectrum disorders. Journal of Intellectual Disability Research.

[CR56] Mandell D (2008). Psychiatric hospitalization among children with autism spectrum disorders. Journal of Autism and Developmental Disorders.

[CR59] Matson JL, Shoemaker M (2009). Intellectual disability and its relationship to autism spectrum disorders. Research in Developmental Disabilities.

[CR58] Matson JL, Baglio CS, Smiroldo BB, Hamilton M, Packlowskyj T, Williams D (1996). Characteristics of autism as assessed by the diagnostic assessment for the severely handicapped-II (DASH-II). Research in Developmental Disabilities.

[CR57] Mannion A, Leader G, Healy O (2013). An investigation of comorbid psychological disorders, sleep problems, gastrointestinal symptoms and epilepsy in children and adolescents with autism spectrum disorder. Research in Autism Spectrum Disorders.

[CR55] Maddox BB, Trubanova A, White SW (2017). Untended wounds: non-suicidal self-injury in adults with autism spectrum disorder. Autism.

[CR60] Matson JL, Turygin NC (2012). How do researchers define behaviour?. Research in Developmental Disabilities.

[CR61] Matson JL, Wilkins J, Macken J (2009). The relationship of challenging behaviours to severity and symptoms of autism spectrum disorders. Journal of Mental Health Research in Intellectual Disabilities.

[CR62] McClintock K, Hall S, Oliver C (2003). Risk markers associated with challenging behaviors in people with intellectual disabilities: A meta-analytic study. Journal of Intellectual Disability Research.

[CR63] McTiernan A, Leader G, Healy O, Mannion A (2011). Analysis of risk factors and early predictors of challenging behaviour for children with autism spectrum disorder. Research in Autism Spectrum Disorders.

[CR64] Minshawi N, Hurwitz S, Fodstad J, Biebl S, Morris D (2014). The association between self-injurious behaviours and autism spectrum disorders. Psychology Research and Behaviour Management.

[CR65] Moher D, Liberati A, Tetzlaff J, Altman DG, The PRISMA Group (2009). Preferred reporting items for systematic reviews and meta-analyses: The PRISMA statement. PLoS Med.

[CR66] Morgan C, Wedd RT, Carr MJ, Kontopantelis E, Green J, Chew-Graham CA (2017). Incidence, clinical management, and mortality risk following self harm among children and adolescents: cohort study in primary care. British Medical Journal.

[CR67] Moyal WN, Lord C, Walkup JT (2014). Quality of life in children and adolescents with autism spectrum disorders: What is known about the effects of pharmacotherapy?. Paediatric Drugs.

[CR68] Murphy O, Healy O, Leader G (2009). Risk factors for challenging behaviors among 157 children with autism spectrum disorders in Ireland. Research in Autism Spectrum Disorders.

[CR69] NICE. (2015). Challenging behaviour and learning disabilities: prevention and interventions for people with learning disabilities whose behaviour challenges. Retrieved from https://www.nice.org.uk/guidance/ng11. 26180881

[CR70] Oliver C, Murphy G, Corbett JA (1987). Self-injurious behaviour in people with mental handicap: A total population study. Journal of Mental Deficiency Research.

[CR71] Oliver C, Richards C (2015). Practitioner Review: Self-injurious behaviour in children with developmental delay. Journal of Child Psychology and Psychiatry.

[CR72] Poustka F, Lisch S (1993). Autistic behaviour domains and their relation to self-injurious behaviour. Acta Paedopsychiatrica.

[CR73] Rattaz C, Michelon C, Baghdadli A (2015). Symptom severity as a risk factor for self-injurious behaviours in adolescents with autism spectrum disorders. Journal of Intellectual Disability Research.

[CR74] Richa S, Fahed M, Khoury E, Mishara B (2014). Suicide in autism spectrum disorders. Arch Suicide Research.

[CR78] Richards C, Davies L, Oliver C (2017). Predictors of self-injurious behaviour and self-restraint in autism spectrum disorder: Towards a hypothesis of impaired behavioural control. Journal of Autism and Developmental Disorders.

[CR76] Richards C, Jones C, Groves L, Moss J, Oliver C (2015). Prevalence of autism spectrum disorder phenomenology in genetic disorders: A systematic review and meta-analysis. Lancet Psychiatry.

[CR77] Richards C, Moss J, Nelson L, Oliver C (2016). Persistence of self-injurious behavior in autism spectrum disorder over 3 years: A prospective cohort study of risk markers. Journal of Neurodevelopmental Disorders.

[CR75] Richards C, Oliver C, Nelson L, Moss J (2012). Self-injurious behaviours in individual with autism and intellectual disability. Journal of Intellectual Disability Research.

[CR79] Richler J, Bishop SL, Kleinke JR, Lord C (2007). Restricted and repetitive behaviours in young children with autism spectrum disorders. Journal of Autism and Developmental Disorders.

[CR80] Saracino J, Noseworthy J, Steiman M, Reisinger L, Fombonne E (2010). Diagnostic and assessment issues in autism surveillance and prevalence. Journal of Developmental and Physical Disabilities.

[CR81] Seltzer M, Greenberg J, Hong J, Smith L, Almeida D, Coe C (2010). Maternal cortisol levels and behavior problems in adolescents and adults with ASD. Journal of Autism and Developmental Disorders.

[CR82] Siegel M, Smith KA, Mazefsky C, Gabriels RL, Erickson C, Kaplan D (2015). The autism inpatient collection: Methods and preliminary sample description. Molecular Autism.

[CR83] Slingsby B, Yatchmink Y, Goldberg A (2017). Typical skin injuries in children with autism spectrum disorder. Clinical Pediatrics.

[CR84] Soke GN, Rosenberg SA, Hamman RF, Fingerlin T, Robinson C, Carpeter L (2016). Brief report: Prevalence of self-injurious behaviors among children with autism spectrum disorder—a population-based study. Journal of Autism Developmental Disorder.

[CR85] Soke GN, Rosenberg SA, Rosenberg CR, Vasa RA, Lee L-C, DiGuiseppi C (2018). Brief report: Self-injurious behaviours in preschool children with autism spectrum disorder compared to other developmental delays and disorders. Journal of Autism and Developmental Disorders.

[CR86] Summers J, Shahrami A, Cali S, D’Mello C, Kako M, Palikucin-Reljin A (2017). Self-injury in autism spectrum disorder and intellectual disability: Exploring the role of reactivity to pain and sensory input. Brain Sciences.

[CR87] Surtees ADR, Oliver C, Jones C, Evans D, Richards C (2018). Shorter duration and poorer quality sleep in people with intellectual disabilities: A meta-analysis. Sleep Medicine Reviews.

[CR88] Victor SE, Klonsky ED (2014). Correlates of suicide attempts among self-injurers: A meta-analysis. Clinical Psychology Review.

[CR89] Weiss J (2002). Self-injurious behaviors in autism: A literature review. Journal on Developmental Disabilities.

[CR90] Woodman AC, Smith LE, Greenberg JS, Mailick MR (2015). Change in autism symptoms and maladaptive behaviours in adolescence and adulthood: The role of positive family processes. Journal of Autism and Developmental Disorders.

