# Supplementary material for: The Prevalence of Self-injurious Behaviour in Autism: A Meta-analytic Study
Source: J Autism Dev Disord. 2020 Apr 15;50(11):3857–73. doi: 10.1007/s10803-020-04443-1 (PMC7557528; doi:10.1007/s10803-020-04443-1)
Supplement: Supplementary file 1 — Supplementary file1 (PDF 13 kb) [file 10803_2020_4443_MOESM1_ESM.pdf]

The Prevalence of Self-Injurious Behaviour in Autism: A Meta-Analytic Study, Journal of Autism and Developmental Disorders

Authors: Dr Catherine Steenfeldt-Kristensen, Dr Chris Jones & Dr Caroline Richards, University of Birmingham

Corresponding Author:

Dr Caroline Richards

School of Psychology University of Birmingham Edgbaston Birmingham B15 2TT UK

c.r.richards@bham.ac.uk

0121 4158098

Supplementary Material 1. Quality Criteria for sample identification, autism assessment, measurement of ID and measurement of SIB/self-harm.

	0<br>Poor	1<br>Adequate	2<br>Good	3<br>Excellent
Sample Identification	Not specified/reported	Single restricted or non-random sample e.g., a specialist clinic or previous research study<br>Single regional sample e.g., one autism school or adult service/group home	Multiple restricted or non-random samples e.g., multi-region specialist clinics<br>National non-random sampling e.g., national parent support groups	Random or total population sample
Assessment of autism	Not specified/reported<br>Clinician judgement only	Screening instrument e.g., SCQ, M-CHAT<br>Clinician judgement against specified diagnostic criteria e.g., DSM-IV or ICD-10	Diagnostic instrument e.g., ADI-R, DISCO, ADOS, 3Di	Consensus from multiple assessments, including at least one diagnostic instrument
Measurement of intellectual disability: IQ	Not specified/reported<br>Clinician judgement only	Parent/carer report<br>Recruited from a specialist ID school	Parent/carer report with a well validated measure	Formal IQ test (e.g. Weschler Intelligence Scale for Children)
Measurement of intellectual disability: adaptive functioning	Not specified/reported<br>Clinician judgement only	Parent/carer report	Parent/carer report with a well validated measure	Formal measure of adaptive functioning (e.g. Vineland Adaptive Behaviour Scales)
Measurement of self-injurious behaviour/self-harm	Not specified/reported	Parent/carer report	Direct observation of behaviour by clinician/researcher OR use of a formal, validated scale (SIB -Q, SIQ, CBQ)	Consensus from multiple assessments including at least one direct observation or formal, validated scale (**SIB -Q, SIQ, CBQ** to assess for presence of SIB /self-harm in combination with direct observation of behaviour
