# Supplementary material for: The Prevalence of Self-injurious Behaviour in Autism: A Meta-analytic Study
Source: J Autism Dev Disord. 2020 Apr 15;50(11):3857–73. doi: 10.1007/s10803-020-04443-1 (PMC7557528; doi:10.1007/s10803-020-04443-1)
Supplement: Supplementary file 2 — Supplementary file2 (PDF 152 kb) [file 10803_2020_4443_MOESM2_ESM.pdf]

The Prevalence of Self-Injurious Behaviour in Autism: A Meta-Analytic Study, Journal of Autism and Developmental Disorders

Authors: Dr Catherine Steenfeldt-Kristensen, Dr Chris Jones & Dr Caroline Richards, University of Birmingham

Corresponding Author:

Dr Caroline Richards

School of Psychology University of Birmingham Edgbaston Birmingham B15 2TT UK

c.r.richards@bham.ac.uk

0121 4158098

Supplementary Material 2. List of papers excluded at eligibility screening through full text review.

1. Adeosun, I. I., Ogun, O. C., Ijarogbe, T., Bello, A. O., Adegbohun, A., & Omigbodun, O. O. (2012). Self-injurious behaviour in Nigerian children with intellectual disability. *Neuropsychiatrie de l'enfance et de l'adolescence*, *60*(5), S170.

2. Berkson, G., Tupa, M., & Sherman, L. (2001). Early development of stereotyped and self-injurious behaviors: I. Incidence. *American Journal on Mental Retardation*, *106*(6), 539-547.

3. Bilenberg, N. (2013). Co-occurring symptoms in a mixed clinical sample of children with autism spectrum disorders. *European Child and Adolescent Psychiatry 22,* S208-S208.

4. Canitano, R. (2006). Self injurious behavior in autism: clinical aspects and treatment with risperidone. *Journal of neural transmission*, *113*(3), 425-431.

5. Carroll, D., Hallett, V., McDougle, C. J., Aman, M. G., McCracken, J. T., Tierney, E., ... & Swiezy, N. (2014). Examination of aggression and self-injury in children with autism spectrum disorders and serious behavioral problems. *Child and Adolescent Psychiatric Clinics*, *23*(1), 57-72.

6. Cervantes, P., Matson, J. L., Tureck, K., & Adams, H. L. (2013). The relationship of comorbid anxiety symptom severity and challenging behaviors in infants and toddlers with autism spectrum disorder. *Research in Autism Spectrum Disorders*, *7*(12), 1528-1534.

7. Cervantes, P. E., & Matson, J. L. (2015). Comorbid symptomology in adults with autism spectrum disorder and intellectual disability. *Journal of autism and developmental disorders*, *45*(12), 3961-3970.

8. Charfi, N., Halayem, S., Touati, M., Mrabet, A., & Bouden, A. (2016). Study of risk factors for aggressive behavior in autism spectrum disorders. *Neuropsychiatrie de l'Enfance et de l'Adolescence*, *64* (3), 147-154.

9. Cohen, I. L., Tsiouris, J. A., Flory, M. J., Kim, S. Y., Freedland, R., Heaney, G., ... & Brown, W. T. (2010). A large scale study of the psychometric characteristics of the IBR modified overt aggression scale: Findings and evidence for increased self-destructive behaviors in adult females with autism spectrum disorder. *Journal of autism and developmental disorders*, *40*(5), 599-609.

10. Collacott, R. A., Cooper, S. A., Branford, D., & McGrother, C. (1998). Epidemiology of self-injurious behaviour in adults with learning disabilities. *The British Journal of Psychiatry*, *173*(5), 428-432.

11. Dempsey, J., Dempsey, A. G., Guffey, D., Minard, C. G., & Goin-Kochel, R. P. (2016). Brief report: further examination of self-injurious behaviors in children and adolescents with autism spectrum disorders. *Journal of autism and developmental disorders*, *46*(5), 1872-1879.

12. Dimian, A. F., Botteron, K. N., Dager, S. R., Elison, J. T., Estes, A. M., Pruett, J. R., ... & Ibis Network. (2017). Potential risk factors for the development of self-injurious behavior among infants at risk for autism spectrum disorder. *Journal of autism and developmental disorders*, *47*(5), 1403-1415.

13. Eden, K. E., de Vries, P. J., Moss, J., Richards, C., & Oliver, C. (2014). Self-injury and aggression in tuberous sclerosis complex: cross syndrome comparison and associated risk markers. *Journal of neurodevelopmental disorders*, *6*(1), 10.

14. Esan, F., Chester, V., Gunaratna, I. J., Hoare, S., & Alexander, R. T. (2015). The clinical, forensic and treatment outcome factors of patients with autism spectrum disorder treated in a forensic intellectual disability service. *Journal of Applied Research in Intellectual Disabilities*, *28*(3), 193-200.

15. Gal, E., Dyck, M. J., & Passmore, A. (2010). Relationships between stereotyped movements and sensory processing disorders in children with and without developmental or sensory disorders. *American Journal of Occupational Therapy*, *64*(3), 453-461.

16. Sankar, U. G. Effect of Sensory Integration Therapy on Self-Stimulating and Self-Injurious Behaviours in Children with Autism: A Pilot study.

17. Griffin, J. C., Ricketts, R. W., Williams, D. E., Locke, B. J., Altmeyer, B. K., & Stark, M. T. (1987). A community survey of self-injurious behavior among developmentally disabled children and adolescents. *Psychiatric Services*, *38*(9), 959-963.

18. Guinchat, V., Cravero, C., Diaz, L., Perisse, D., Xavier, J., Amiet, C., ... & Consoli, A. (2015). Acute behavioral crises in psychiatric inpatients with autism spectrum disorder (ASD): recognition of concomitant medical or non-ASD psychiatric conditions predicts enhanced improvement. *Research in Developmental Disabilities*, *38*, 242-255.

19. Hoch, J., Spofford, L., Dimian, A., Tervo, R., MacLean, W. E., & Symons, F. J. (2016). A direct comparison of self-injurious and stereotyped motor behavior between preschool-aged children with and without developmental delays. *Journal of pediatric psychology*, *41*(5), 566-572.

20. Ianni, H. F., Abreu, T. C., Fidelis, S. D. M., Correa, H., & Kummer, A. (2015). Prevalence of self-injurious behavior in people with intellectual development disorder. *Brazilian Journal of Psychiatry*, *37*(3), 266-267.

21. Jiang Z., Li, J., Guo, L., & Tan, L. (2013). A comparative study with autism and mental retardation children about repetitive behavior: 41. *Developmental Medicine & Child Neurology*, *55*.

22. Kerns, C. M., Kendall, P. C., Zickgraf, H., Franklin, M. E., Miller, J., & Herrington, J. (2015). Not to be overshadowed or overlooked: Functional impairments associated with comorbid anxiety disorders in youth with ASD. *Behavior therapy*, *46*(1), 29-39.

23. Kesic, A., Lakic, A., Ninkovic, M., & Markovic, J. (2015). Risperidone use in children and adolescents with autism spectrum disorders and problematic behavior. *European Child and Adolescent Psychiatry 24,* S164-S164.

24. Kolevzon, A., Lim, T., Schmeidler, J., Martello, T., Cook Jr, E. H., & Silverman, J. M. (2014). Self-injury in autism spectrum disorder: An effect of serotonin transporter gene promoter variants. *Psychiatry research*, *220*(3), 987-990.

25. Koshy, B., Maskey, M., Warnell, F., Johnson, M., Mcconachie, H., Le Couteur, A., & Parr, J. (2013). ASD+ Study-co-existing conditions in children with ASD: evidence from two large UK databases: 17. *Developmental Medicine & Child Neurology*, *55*.

26. Kurtz, P. F., Chin, M. D., Huete, J. M., Tarbox, R. S., O'Connor, J. T., Paclawskyj, T. R., & Rush, K. S. (2003). Functional analysis and treatment of self-injurious behavior in young children: A summary of 30 cases. *Journal of applied behavior analysis*, *36*(2), 205-219.

27. Lance, E., York, J., Lee, L. C., & Zimmerman, A. W. (2011). Association between regression and self injurious behaviors among children with ASD. *Annals of Neurology, 70,* S138-S138.

28. Lance, E. I., York, J. M., Lee, L. C., & Zimmerman, A. W. (2014). Association between regression and self injury among children with autism. *Research in developmental disabilities*, *35*(2), 408-413.

29. MacLean Jr, W. E., Tervo, R. C., Hoch, J., Tervo, M., & Symons, F. J. (2010). Self-injury among a community cohort of young children at risk for intellectual and developmental disabilities. *The Journal of Pediatrics*, *157*(6), 979-983.

30. MacLean, W. E., & Dornbush, K. (2012). Self-injury in a statewide sample of young children with developmental disabilities. *Journal of Mental Health Research in Intellectual Disabilities*, *5*(3-4), 236-245.

31. Matson, J. L., Boisjoli, J., & Mahan, S. (2009). The relation of communication and challenging behaviors in infants and toddlers with autism spectrum disorders. *Journal of Developmental and Physical Disabilities*, *21*(4), 253-261.

32. Matson, J. L., Hamilton, M., Duncan, D., Bamburg, J., Smiroldo, B., Anderson, S., ... & Kirkpatrick-Sanchez, S. (1997). Characteristics of stereotypic movement disorder and self-injurious behavior assessed with the Diagnostic Assessment for the Severely Handicapped (DASH-II). *Research in Developmental Disabilities*, *18*(6), 457-469.

33. Matson, J. L., Mahan, S., Fodstad, J. C., Worley, J. A., Neal, D., & Sipes, M. (2011). Effects of symptoms of co-morbid psychopathology on challenging behaviours among infants and toddlers with Autistic Disorder and PDD-NOS as assessed with the Baby and Infant Screen for Children with aUtIsm Traits (BISCUIT). *Developmental neurorehabilitation*, *14*(3), 129-139.

34. Matson, J. L., Neal, D., Fodstad, J. C., & Hess, J. A. (2010). The relation of social behaviours and challenging behaviours in infants and toddlers with autism spectrum disorders. *Developmental Neurorehabilitation*, *13*(3), 164-169.

35. Matson, J. L., & Rivet, T. T. (2008). The effects of severity of autism and PDD-NOS symptoms on challenging behaviors in adults with intellectual disabilities. *Journal of Developmental and Physical Disabilities*, *20*(1), 41-51.

36. Research Units on Pediatric Psychopharmacology Autism Network. (2005). Risperidone treatment of autistic disorder: longer-term benefits and blinded discontinuation after 6 months. *American Journal of Psychiatry*, *162*(7), 1361-1369.

37. Mehl-Madrona, L., Leung, B., Kennedy, C., Paul, S., & Kaplan, B. J. (2010). Micronutrients versus standard medication management in autism: a naturalistic case–control study. *Journal of child and adolescent psychopharmacology*, *20*(2), 95-103.

38. Ming, X., Brimacombe, M., Chaaban, J., Zimmerman-Bier, B., & Wagner, G. C. (2008). Autism spectrum disorders: concurrent clinical disorders. *Journal of child neurology*, *23*(1), 6-13.

39. Murphy, G., Hall, S., Oliver, C., & Kissi-Debra, R. (1999). Identification of early self-injurious behaviour in young children with intellectual disability. *Journal of Intellectual Disability Research*, *43*(3), 149-163.

40. Poppes, P., Van der Putten, A. J. J., & Vlaskamp, C. (2010). Frequency and severity of challenging behaviour in people with profound intellectual and multiple disabilities. *Research in developmental disabilities*, *31*(6), 1269-1275.

41. Posey, D. J., Guenin, K. D., Kohn, A. E., Swiezy, N. B., & McDougle, C. J. (2001). A naturalistic open-label study of mirtazapine in autistic and other pervasive developmental disorders. *Journal of child and adolescent psychopharmacology*, *11*(3), 267-277.

42. Richards, C., Moss, J., Nelson, L., & Oliver, C. (2016). Persistence of self-injurious behaviour in autism spectrum disorder over 3 years: a prospective cohort study of risk markers. *Journal of neurodevelopmental disorders*, *8*(1), 21.

43. Rojahn, J., Wilkins, J., Matson, J. L., & Boisjoli, J. (2010). A comparison of adults with intellectual disabilities with and without ASD on parallel measures of challenging behaviour: The Behavior Problems Inventory-01 (BPI-01) and Autism Spectrum Disorders-Behavior Problems for Intellectually Disabled Adults (ASD-BPA). *Journal of Applied Research in Intellectual Disabilities*, *23*(2), 179-185.

44. Shahwan, S. (2016). Moderate to severe self-injury among adolescents seeking treatment in IMH. *Annals of the Academy of Medicine Singapore 45* (9), S98.

45. Smith, K. R., & Matson, J. L. (2010). Behavior problems: Differences among intellectually disabled adults with co-morbid autism spectrum disorders and epilepsy. *Research in Developmental Disabilities*, *31*(5), 1062-1069.

46. Soke, G. N., Rosenberg, S. A., Hamman, R. F., Fingerlin, T., Rosenberg, C. R., Carpenter, L., ... & Reynolds, A. (2017). Factors associated with self-injurious behaviors in children with autism spectrum disorder: findings from two large national samples. *Journal of autism and developmental disorders*, *47*(2), 285-296.

47. Summers, J., Shahrami, A., Cali, S., D'Mello, C., Kako, M., Palikucin-Reljin, A., ... & Lunsky, Y. (2017). Self-injury in autism spectrum disorder and intellectual disability: exploring the role of reactivity to pain and sensory input. *Brain sciences*, *7*(11), 140.

48. Symons, F. J., & Thompson, T. (1997). Self-injurious behaviour and body site preference. *Journal of Intellectual Disability Research*, *41*(6), 456-468.

49. Tureck, K., Matson, J. L., & Beighley, J. S. (2013). An investigation of self-injurious behaviors in adults with severe intellectual disabilities. *Research in developmental disabilities*, *34*(9), 2469-2474.

50. Williams, D. L., Siegel, M., & Mazefsky, C. A. (2018). Problem behaviors in autism spectrum disorder: Association with verbal ability and adapting/coping skills. *Journal of autism and developmental disorders*, *48*(11), 3668-3677.

51. Wolff, J. J., Hazlett, H. C., Lightbody, A. A., Reiss, A. L., & Piven, J. (2013). Repetitive and self-injurious behaviors: associations with caudate volume in autism and fragile X syndrome. *Journal of neurodevelopmental disorders*, *5*(1), 12.

52. Yang, Y., Kim, Y., Won, E., Bahn, G., Choi, J., Han, J., ... & Jung, M. (2015). Is self-injurious behavior more frequent in autism spectrum disorder individuals with aggressive behaviors than in those without aggressive behaviors? Preliminary study. *European Child and Adolescent Psychiatry*, *24*, S203-S204.
