# Supplementary material for: The Prevalence of Self-injurious Behaviour in Autism: A Meta-analytic Study
Source: J Autism Dev Disord. 2020 Apr 15;50(11):3857–73. doi: 10.1007/s10803-020-04443-1 (PMC7557528; doi:10.1007/s10803-020-04443-1)
Supplement: Supplementary file 3 — Supplementary file3 (PDF 893 kb) [file 10803_2020_4443_MOESM3_ESM.pdf]

The Prevalence of Self-Injury in Autism: A Meta-Analytic Review, Journal of Autism and Developmental Disorders

Authors: Dr Catherine Steenfeldt-Kristensen, Dr Chris A. Jones & Dr Caroline Richards, University of Birmingham

Corresponding Author:

Dr Caroline Richards

School of Psychology
University of Birmingham
Edgbaston
Birmingham
B15 2TT
UK
c.r.richards@bham.ac.uk

0121 4158098

Supplementary Materials 3: Tables and Forest Plots of individual topographies of self-injurious behaviour

Table 9 Reporting of the prevalence rates and confidence intervals for hair pulling**.**

Study Name	PR	95%-CI	Weighting in Random Effects Model
**Akram 2017**	0.1	[0.0412; 0.1588]	7.9
**Buono 2010**	0.06	[0.0012; 0.1188]	7.9
**Duerden 2012**	0.19	[0.1312; 0.2488]	7.9
**Folch 2018**	0.06	[0.0208; 0.0992]	8.6
**Gulsrud 2018**	0.01	[-0.0096; 0.0296]	9.2
**Handen 2018**	0.25	[0.2108; 0.2892]	8.6
**Kamio 2002**	0.02	[0.0004; 0.0396]	9.2
**Maddox 2017**	0.12	[0.0220; 0.2180]	6.1
**Richards 2012**	0.06	[0.0208; 0.0992]	8.6
**Richards 2017 Child**	0.09	[0.0508; 0.1292]	8.6
**Richards 2017 Adult**	0.1	[0.0608; 0.1392]	8.6
**Slingsby 2017**	0.02	[-0.0192; 0.0592]	8.6

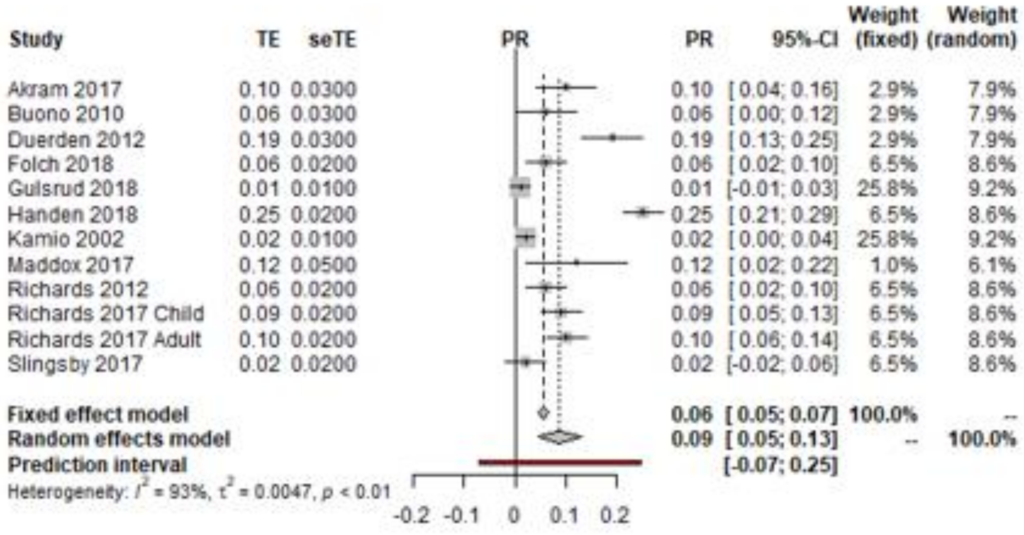

Fig. 6 The pooled prevalence estimates for hair pulling in autism using the random-effects model

Table 10 Reporting of the prevalence rates and confidence intervals for hand hitting.

Study Name	PR	95%-CI	Weighting in Random Effects Model
**Akram 2017**	0.16	[0.0816; 0.2384]	7.1
**Buono 2010**	0.41	[0.2728; 0.5472]	6
**Dominick 2007**	0.17	[0.0720; 0.2680]	6.8
**Duerden 2012**	0.34	[0.2616; 0.4184]	7.1
**Folch 2018**	0.32	[0.2416; 0.3984]	7.1
**Gulsrud 2018**	0.09	[0.0508; 0.1292]	7.6
**Handen 2018**	0.48	[0.4212; 0.5388]	7.4
**Hattier 2011**	0.06	[0.0404; 0.0796]	7.8
**Lecavalier 2006**	0.16	[0.1208; 0.1992]	7.6
**Maddox 2017**	0.12	[0.0220; 0.2180]	6.8
**Richards 2012**	0.15	[0.0912; 0.2088]	7.4
**Richards 2017 Child**	0.25	[0.1912; 0.3088]	7.4
**Richards 2017 Adult**	0.28	[0.2212; 0.3388]	7.4
**Slingsby 2017**	0.22	[0.1024; 0.3376]	6.4

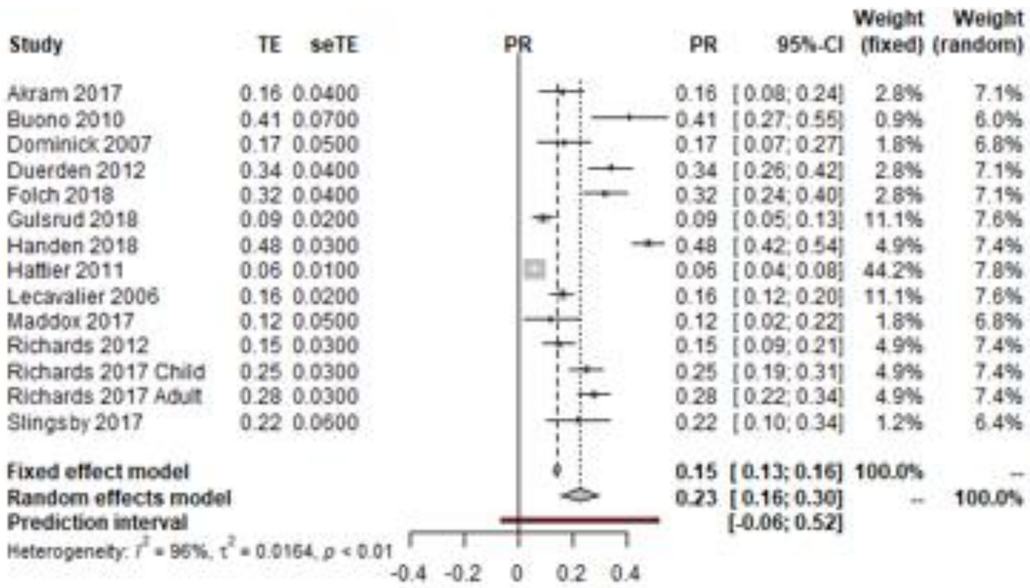

Fig. 7 The pooled prevalence estimates for hand hitting in autism using the random-effects model

Table 11. Reporting of the prevalence rates and confidence intervals for head banging.

Study Name	PR	95%-CI	Weighting in Random Effects Model
**Dominick 2007**	0.2200	[0.1024; 0.3376]	14.3
**Folch 2018**	0.1000	[0.0608; 0.1392]	49.9
**Gulsrud 2018**	0.1000	[0.0412; 0.1588]	35.9

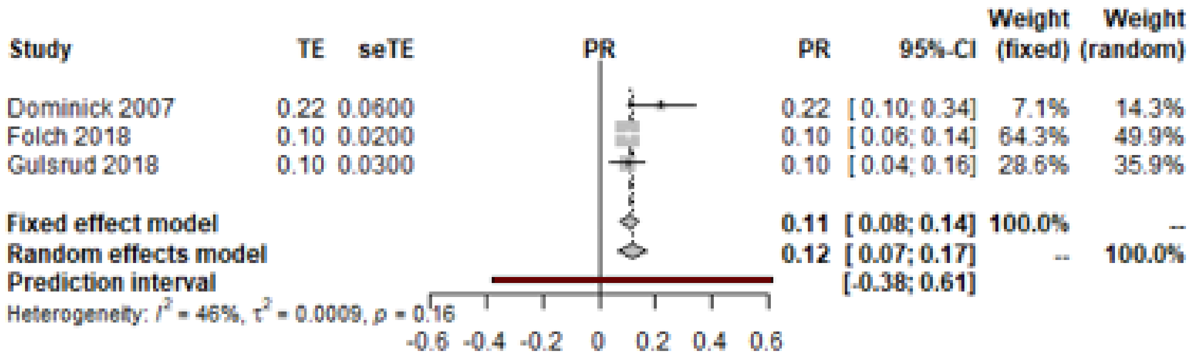

Fig. 8 The pooled prevalence estimates for head banging in autism using the random-effects model

Table 12. Reporting of the prevalence rates and confidence intervals for hitting self against objects.

Study Name	PR	95%-CI %	Weighting in Random Effects Model
**Duerden 2012**	0.3	[0.2412; 0.3588]	14.2
**Handen 2018**	0.47	[0.4112; 0.5288]	14.2
**Hattier 2011**	0.09	[0.0704; 0.1096]	14.9
**Richards 2012**	0.08	[0.0408; 0.1192]	14.6
**Richards 2017 Child**	0.16	[0.1012; 0.2188]	14.2
**Richards 2017 Adult**	0.16	[0.1012; 0.2188]	14.2
**Slingsby 2017**	0.07	[-0.0084; 0.1484]	13.6

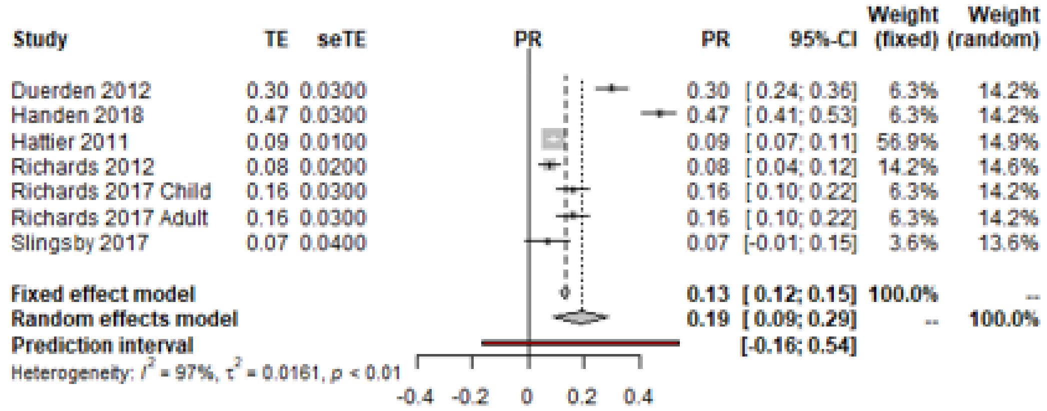

Fig. 9 The pooled prevalence estimates for hitting self against objects in autism using the random-effects model

Table 13. Reporting of the prevalence rates and confidence intervals for object hitting.

Study Name	PR	95%-CI %W	Weighting in Random Effects Model
**Buono 2010**	0.41	[0.2728; 0.5472]	9.1
**Duerden 2012**	0.18	[0.1212; 0.2388]	13.2
**Handen 2018**	0.22	[0.1808; 0.2592]	14
**Maddox 2017**	0.14	[0.0420; 0.2380]	11.2
**Richards 2012**	0.05	[0.0108; 0.0892]	14
**Richards 2017 Child**	0.06	[0.0208; 0.0992]	14
**Richards 2017 Adult**	0.03	[0.0104; 0.0496]	14.5
**Slingsby 2017**	0.17	[0.0524; 0.2876]	10.1

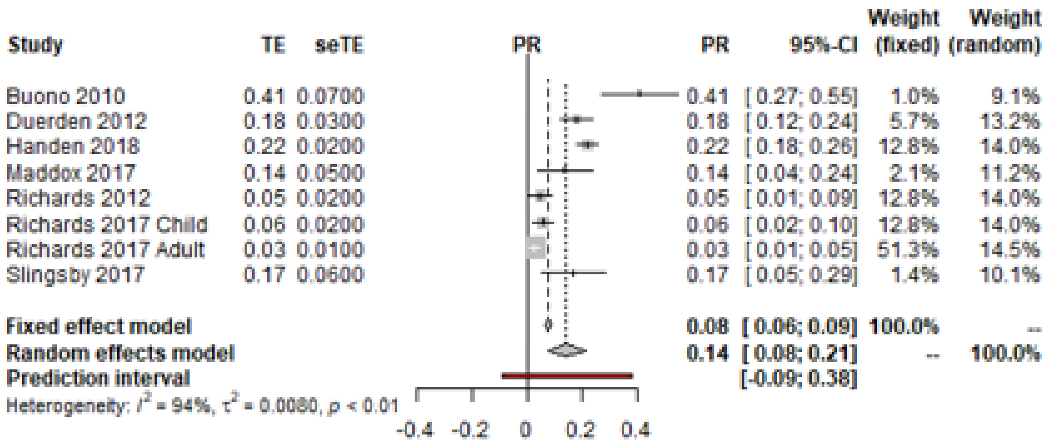

Fig. 10 The pooled prevalence estimates for object hitting in autism using the random-effects model

Table 14. Reporting of the prevalence rates and confidence intervals for inserting objects in cavities.

Study Name	PR	95%-CI	Weighting in Random Effects Model
**Buono 2010**	0.35	[0.2128; 0.4872]	3.8
**Duerden 2012**	0.18	[0.1212; 0.2388]	9.3
**Folch 2018**	0.01	[-0.0096; 0.0296]	13.2
**Handen 2018**	0.11	[0.0708; 0.1492]	11.4
**Hattier 2011**	0.02	[0.0004; 0.0396]	13.2
**Lecavalier 2006**	0.11	[0.0708; 0.1492]	11.4
**Richards 2012**	0.03	[0.0104; 0.0496]	13.2
**Richards 2017 Child**	0.06	[0.0208; 0.0992]	11.4
**Richards 2017 Adult**	0.05	[0.0304; 0.0696]	13.2

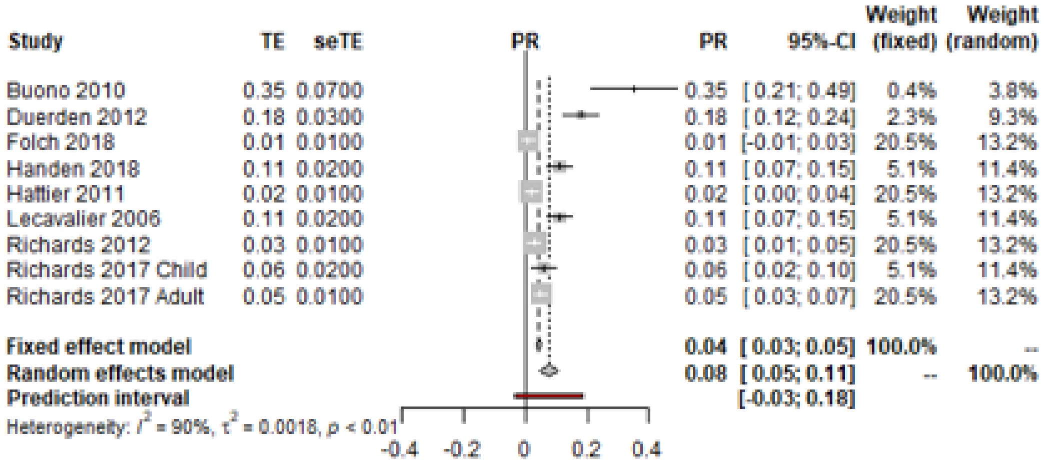

Fig. 11 The pooled prevalence estimates for inserting objects in cavities in autism using the random-effects model

Table 15. Reporting of the prevalence rates and confidence intervals for rubbing self on surfaces.

Study Name	PR	95%-CI	Weighting in Random Effects Model
**Akram 2017**	0.0600	[0.0012; 0.1188]	73.5
**Maddox 2017**	0.01000	[0.0020; 0.1980]	26.5

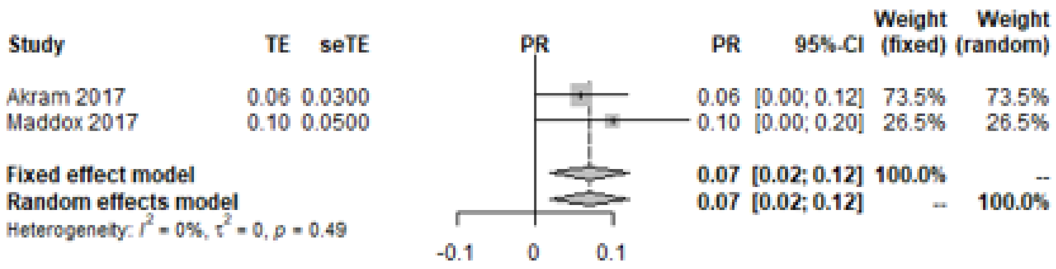

Fig. 12 The pooled prevalence estimates for rubbing self on surfaces in autism using the random-effects model

Table 16. Reporting of the prevalence rates and confidence intervals for self-biting.

Study Name	PR	95%-CI	Weighting in Random Effects Model
**Akram 2017**	0.11	[0.0512; 0.1688]	8.5
**Buono 2010**	0.39	[0.2528; 0.5272]	5.6
**Dominick 2007**	0.09	[0.0116; 0.1684]	7.8
**Duerden 2012**	0.26	[0.2012; 0.3188]	8.5
**Gulsrud 2018**	0.03	[-0.0092; 0.0692]	9.1
**Handen 2018**	0.3	[0.2412; 0.3588]	8.5
**Kamio 2002**	0.02	[0.0004; 0.0396]	9.5
**Lecavalier 2006**	0.05	[0.0304; 0.0696]	9.5
**Richards 2012**	0.09	[0.0508; 0.1292]	9.1
**Richards 2017 Child**	0.17	[0.1112; 0.2288]	8.5
**Richards 2017 Adult**	0.16	[0.1208; 0.1992]	9.1
**Slingsby 2017**	0.17	[0.0524; 0.2876]	6.3

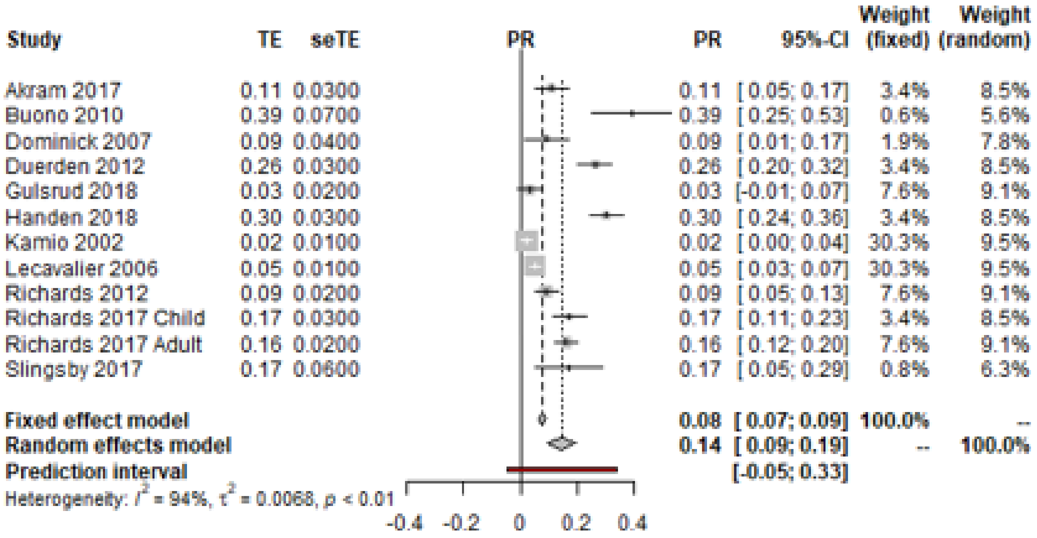

Fig. 13 The pooled prevalence estimates for self-biting in autism using the random-effects model

Table 17. Reporting of the prevalence rates and confidence intervals for self-pinching.

Study Name	PR	95%-CI	Weighting in Random Effects Model
**Akram 2017**	0.1100	[0.0512; 0.1688]	15.7
**Buono 2010**	0.0600	[0.0012; 0.1188]	15.7
**Hattier 2011**	0.0600	[0.0404; 0.0796]	68.6

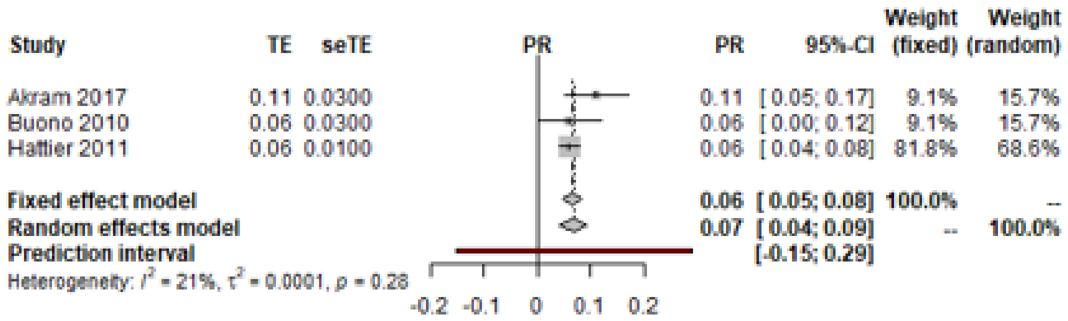

Fig. 14 The pooled prevalence estimates for self-pinching in autism using the random-effects model

Study Name	PR	95%-CI %W	Weighting in Random Effects Model
**Akram 2017**	0.12	[0.0416; 0.1984]	6.9
**Buono 2010**	0.14	[0.0420; 0.2380]	6.2
**Duerden 2012**	0.23	[0.1712; 0.2888]	7.6
**Folch 2018**	0.15	[0.0912; 0.2088]	7.6
**Gulsrud 2018**	0.01	[-0.0096; 0.0296]	8.6
**Handen 2018**	0.43	[0.3712; 0.4888]	7.6
**Hattier 2011**	0.06	[0.0404; 0.0796]	8.6
**Kamio 2002**	0.04	[0.0204; 0.0596]	8.6
**Lecavalier 2006**	0.1	[0.0804; 0.1196]	8.6
**Maddox 2017**	0.36	[0.2228; 0.4972]	4.8
**Richards 2012**	0.09	[0.0508; 0.1292]	8.2
**Richards 2017 Child**	0.12	[0.0808; 0.1592]	8.2
**Richards 2017 Adult**	0.15	[0.1108; 0.1892]	8.2

Table 18. Reporting of the prevalence rates and confidence intervals for self-scratching.

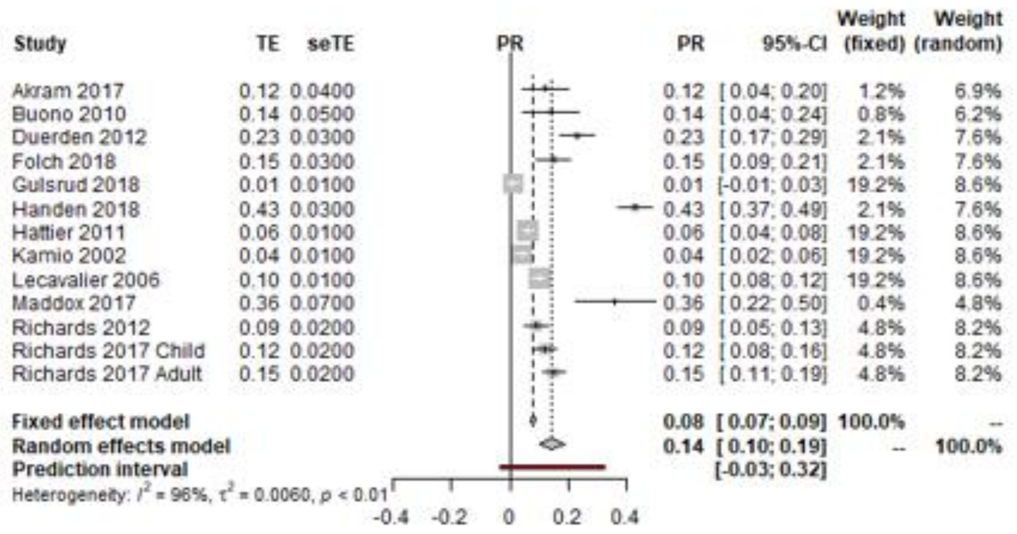

Fig. 15 The pooled prevalence estimates for self-scratching in autism using the random-effects model

Table 19. Reporting of the prevalence rates and confidence intervals for wound interfering.

Study Name	PR	95%-CI %	Weighting in Random Effects Model
**Akram 2017**	0.11	[0.0512; 0.1688]	27.7
**Folch 2018**	0.08	[0.0408; 0.1192]	62.3
**Maddox 2017**	0.12	[0.0220; 0.2180]	10

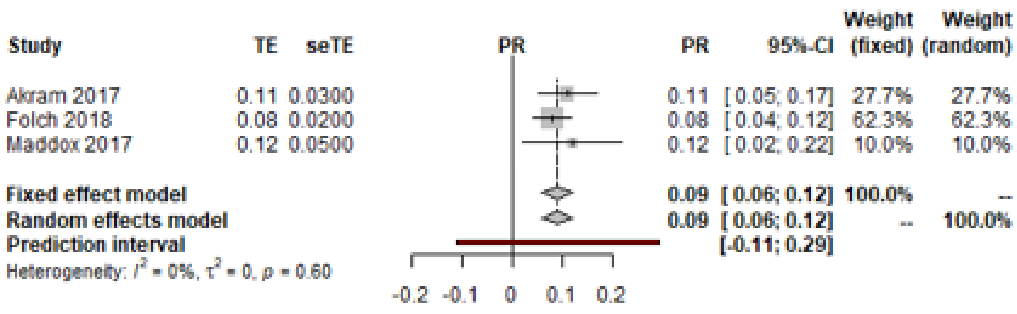

Fig. 16 The pooled prevalence estimates for wound interfering in autism using the random-effects model

Table 20. Reporting of the prevalence rates and confidence intervals for self-cutting.

Study Name	PR	95%-CI %	Weighting in Random Effects Model
**Akram 2017**	0.01	[-0.0096; 0.0296]	24.4
**Folch 2018**	0.01	[-0.0096; 0.0296]	24.4
**Maddox 2017**	0.17	[0.0524; 0.2876]	2.4
**Richards 2017 Child**	0.04	[0.0204; 0.0596]	24.4
**Richards 2017 Adult**	0.03	[0.0104; 0.0496]	24.4

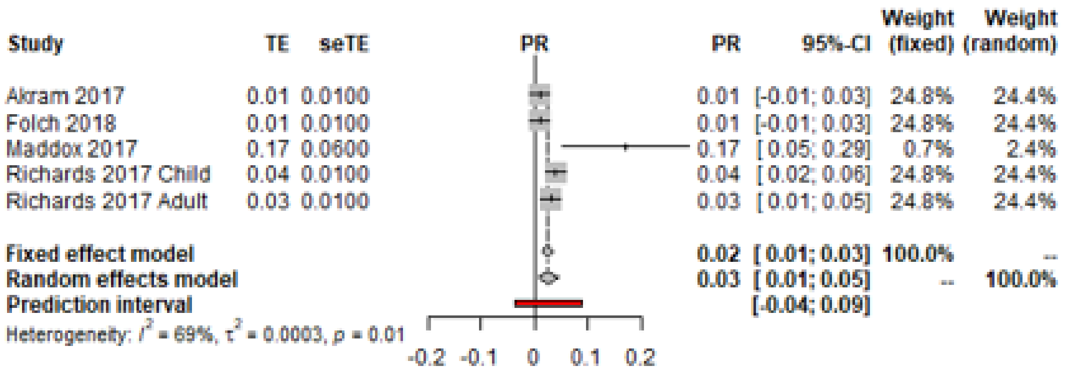

Fig. 17 The pooled prevalence estimates for self-cutting in autism using the random-effects model

Table 21. Reporting of the prevalence rates and confidence intervals for skin picking.

Study Name	PR	95%-CI	Weighting in Random Effects Model
**Duerden 2012**	**0.25**	**[0.1912; 0.3088]**	**33.1**
**Gulsrud 2018**	**0.02**	**[0.0004; 0.0396]**	**33.8**
**Handen 2018**	**0.33**	**[0.2712; 0.3888]**	**33.1**

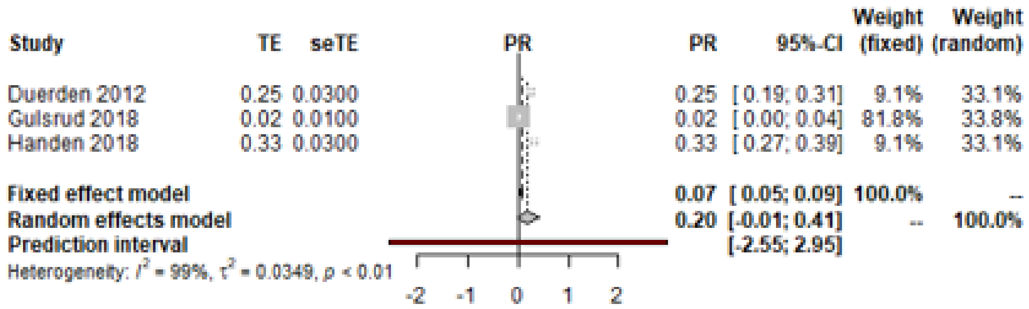

Fig. 18 The pooled prevalence estimates for skin picking in autism using the random-effects model
